# Diesel-derived PM_2.5_ induces impairment of cardiac movement followed by mitochondria dysfunction in cardiomyocytes

**DOI:** 10.3389/fendo.2022.999475

**Published:** 2022-09-28

**Authors:** Tae Hwan Shin, Seok Gi Kim, Moongi Ji, Do Hyeon Kwon, Ji Su Hwang, Nimisha Pradeep George, Dube Solomon Ergando, Chan Bae Park, Man Jeong Paik, Gwang Lee

**Affiliations:** ^1^ Department of Physiology, Ajou University School of Medicine, Suwon, South Korea; ^2^ Department of Molecular Science and Technology, Ajou University, Suwon, South Korea; ^3^ College of Pharmacy, Sunchon National University, Suncheon, South Korea

**Keywords:** diesel particulate matter, mitochondria, cardiomyocytes, metabolomics, transcriptomics, integrated omics

## Abstract

Particulate matter (PM) in polluted air can be exposed to the human body through inhalation, ingestion, and skin contact, accumulating in various organs throughout the body. Organ accumulation of PM is a growing health concern, particularly in the cardiovascular system. PM emissions are formed in the air by solid particles, liquid droplets, and fuel – particularly diesel – combustion. PM_2.5_ (size < 2.5 μm particle) is a major risk factor for approximately 200,000 premature deaths annually caused by air pollution. This study assessed the deleterious effects of diesel-derived PM_2.5_ exposure in HL-1 mouse cardiomyocyte cell lines. The PM_2.5_-induced biological changes, including ultrastructure, intracellular reactive oxygen species (ROS) generation, viability, and intracellular ATP levels, were analyzed. Moreover, we analyzed changes in transcriptomics using RNA sequencing and metabolomics using gas chromatography-tandem mass spectrometry (GC-MS/MS) and liquid chromatography-tandem mass spectrometry (LC-MS/MS) in PM_2.5_-treated HL-1 cells. Ultrastructural analysis using transmission electron microscopy revealed disruption of mitochondrial cristae structures in a PM_2.5_ dose-dependent manner. The elevation of ROS levels and reduction in cell viability and ATP levels were similarly observed in a PM_2.5_ dose-dependently. In addition, 6,005 genes were differentially expressed (fold change cut-off ± 4) from a total of 45,777 identified genes, and 20 amino acids (AAs) were differentially expressed (fold change cut-off ± 1.2) from a total of 28 identified AAs profiles. Using bioinformatic analysis with ingenuity pathway analysis (IPA) software, we found that the changes in the transcriptome and metabolome are highly related to changes in biological functions, including homeostasis of Ca^2+^, depolarization of mitochondria, the function of mitochondria, synthesis of ATP, and cardiomyopathy. Moreover, an integrated single omics network was constructed by combining the transcriptome and the metabolome. *In silico* prediction analysis with IPA predicted that upregulation of mitochondria depolarization, ROS generation, cardiomyopathy, suppression of Ca^2+^ homeostasis, mitochondrial function, and ATP synthesis occurred in PM_2.5_-treated HL-1 cells. In particular, the cardiac movement of HL-1 was significantly reduced after PM_2.5_ treatment. In conclusion, our results assessed the harmful effects of PM_2.5_ on mitochondrial function and analyzed the biological changes related to cardiac movement, which is potentially associated with cardiovascular diseases.

## Introduction

Air pollution has become a major global health problem. Through inhalation, ingestion, and skin contact, it has led to the accumulation of particulate matter (PM) in various organs throughout the body (1). Emissions of particulate matter from fossil fuels – particularly diesel – are a major source of air pollution (2). The toxicological evaluation of fine (size < 2.5 μm) particulate matter (PM_2.5_) has been conducted in the epidermal (2), respiratory (3), immune (4), nervous (5), and cardiovascular systems (6). The cardiovascular system in particular is affected by PM_2.5_. As such, PM_2.5_ is a crucial risk factor for approximately 200,000 premature deaths annually owing to its effect on vascular dysfunction, atherosclerosis, autonomic dysfunction, and hypertension (7). Oxidative stress has been reported to be one of the major factors in PM_2.5_-induced toxicity (7, 8); however, the underlying mechanisms in the cardiovascular system are still not fully understood.

Mitochondria are the major target of oxidative stress. They are highly developed in cardiac muscle cells compared to other cell types (9, 10), occupying over 40% of the spatial portion of the cells. They generate over 90% of ATP in the cardiac muscle cells through aerobic respiration (9). Calcium homeostasis and regulation are particularly important for the proper functioning of the mitochondria (11, 12) and are highly related to the cardiac contraction machinery and mitochondrial polarization (11, 13). As such, mitochondria, as well as calcium homeostasis, are the major targets for PM_2.5_-induced toxicity; however, the underlying biological relationship between them remains poorly understood.

Comprehensive analyses using omics technology have been applied for the toxicological assessment of nanomaterials, polystyrene particles, and urban PM (14–21). Fundamentally, the toxicities of these particles, including PM_2.5_, occur non-specifically and disturb various biological homeostasis simultaneously.

Several limitations in conventional toxicological evaluations have been previously documented (20, 22, 23). Integrated analysis of multi-layer molecules such as genes, proteins, and metabolites has been suggested to overcome these limitations and better understand the complex biological changes. This method is called systems toxicology (24). Moreover, integrated omics analysis can synergize the strengths of single omics analysis and overcome their weaknesses (20–22). In this study, we evaluated the toxicological mechanism of PM_2.5_-induced toxicity in the mitochondria of cardiomyocytes using transcriptomics, metabolomics, and integrated omics analysis.

## Materials and methods

### PM_2.5_ preparation and treatment

Diesel particulate matter (NIST^®^ SRM^®^ 1650b, Sigma-Aldrich, St. Louis, MO, USA) was diluted in dimethyl sulfoxide (DMSO) (10 mg/mL) to form a stock solution and was stored at -20°C. Before use, the stock solution was sonicated for 30 min to prevent particle aggregation (2). The stock solution was then diluted in a Claycomb medium (Sigma-Aldrich) for treatment. Cells were each treated with different concentrations of PM_2.5_ for 12 h. After treatment, each well was washed twice with PBS to reduce the effect of the remaining particles.

### Cell culture

Culture flasks and plates were coated with a 0.02% gelatin (Sigma-Aldrich) and 5 μg/mL fibronectin (Sigma-Aldrich) extracellular matrix (ECM) solution before seeding the cells. HL-1, a murine cardiomyocyte cell line (Merck, Darmstadt, Germany), was cultured in a Claycomb medium supplemented with 10% fetal bovine serum (FBS; Corning, NY, USA), 0.1 mM norepinephrine (Sigma-Aldrich), 2 mM L-glutamine (Sigma-Aldrich), and 100 U/mL penicillin-streptomycin (Thermo Fisher Scientific, Waltham, MA, USA) at 37°C and 5% CO_2_ in a humidified atmosphere. The culture medium was replaced daily.

### Cell viability

Cell viability was measured using a 3-(4,5-Dimethylthiazol-2-yl)-2,5-diphenyltetrazolium bromide (MTT) Cell Proliferation Kit I (Roche, Basel, Switzerland). HL-1 cells were treated with 0, 10, 20, 50, 100, or 200 μg/mL PM_2.5_ for 12 h, followed by treatment with an MTT reagent for 4 h, and then the MTT formazan was dissolved using the solubilization solution provided in the kit and left overnight. Absorbance was measured at 590 nm using a microplate spectrophotometer (Epoch 2; BioTek, Winooski, VT, USA). The influence of DMSO on HL-1 cells was not detected in 0, 0.1, 0.5, 1.0, and 2.0% DMSO, respectively (data not shown).

### Measurement of intracellular ATP level

Relative levels of intracellular ATP were measured using the CellTiter-Glo^®^ luminescent cell viability assay as in previous study (Promega, Madison, WI, USA) (25). The 0, 10, and 100 μg/mL PM_2.5_-treated HL-1 cells were incubated at room temperature (RT) for 30 min, and assay reagent was added equally to the volume of the culture medium. The contents were mixed for 2 min and incubated for 10 min at RT. Luminescence signals were detected using a multi-mode microplate reader (SpectraMax^®^ iD3; Molecular Devices, San Jose, CA, USA).

### Measurement of ROS level

Intracellular ROS levels were measured using the ROS-Glo™ H_2_O_2_ assay (Promega). After PM_2.5_ treatment (0, 10, and 100 μg/mL) for 6 h, H_2_O_2_ substrate was added and incubated for another 6 h at 37°C. The detection solution was added to each well and incubated for 20 min at RT. Luminescence signals were detected using a multi-mode microplate reader (SpectraMax^®^ iD3).

### RNA sequencing and data processing

HL-1 cells were treated with 10 and 100 µg/mL PM_2.5_ for 12 h and then lysed. Total RNA was isolated using QIAzol^®^ (Qiagen, Valencia, CA, USA). Total RNA concentration was quantified using Quant-IT RiboGreen (Invitrogen, Waltham, MA, USA). The integrity of total RNA was determined using TapeStation RNA screentape (Agilent Technologies, Atlanta, GA, USA), and samples with RNA integrity numbers higher than 7 were used for library generation. A library was generated with 1 µg of total RNA from each sample using the Illumina TruSeq Stranded mRNA Sample Prep Kit (Illumina Inc., San Diego, CA, USA). First-strand cDNA was synthesized using SuperScript II reverse transcriptase (Invitrogen). Second-strand cDNA was synthesized using DNA polymerase I, RNase H, and dUTP. The cDNA samples were processed *via* end repair by adding a single “A” base and ligating the adapters. The library samples were analyzed using Illumina NovaSeq (Illumina Inc.).

Raw reads from the sequencer were preprocessed to eliminate low-quality and adapter sequences before analysis, and the processed reads were aligned to the *Mus musculus (mm10)* genome using HISAT v2.1.0 (26). Transcript assembly and abundance estimations were performed using StringTie (27, 28). The aligned reads were assembled, and gene abundance was determined using StringTie v2.1.3b. Biological functions were investigated using bioinformatics ingenuity pathway analysis (IPA, http://www.ingenuity.com) software (Qiagen) (29). A 4-fold expression change for the transcriptome and a 1.2-fold expression change for AA levels were used as cut-off values for significant changes.

### Gas chromatography-tandem mass spectrometry

Profiling analysis of AAs in HL-1 cells was performed using ethoxycarbonylation (EOC)/*tert*-butyldimethylsilyl (TBDMS) derivatives as previously described (30, 31). Briefly, deproteinization was performed by adding acetonitrile (100 μL) to lysed cardiomyocytes (1.0 × 10^6^) containing norvaline as an internal standard (IS; 0.2 µg). After centrifugation, the supernatants were spiked into deionized water (1.0 mL) and added to dichloromethane (2.0 mL) containing ethyl chloroformate (ECF) adjusted to pH ≥ 12 using 5 M sodium hydroxide. A two-phase EOC reaction was performed by vortex mixing for 10 min. After the EOC reaction, the aqueous phase was adjusted to pH ≤ 2 using 10% H_2_SO_4_, saturated with sodium chloride, and sequentially extracted using diethyl ether (3.0 mL) and ethyl acetate (2.0 mL). The extracts were evaporated to dryness under a gentle stream of nitrogen (40°C). Before GC-MS/MS analysis, toluene (20 μL), *N*-Methyl-*N*-*tert*-butyldimethylsilyl trifluoroacetamide (MTBSTFA) (15 μL), and triethylamine (TEA) were added to the residue and then heated at 60°C for 60 min to generate a TBDMS derivative.

GC-MS/MS analysis was performed using a Shimadzu TQ 8040 triple quadruple mass spectrometer (Shimadzu, Kyoto, Japan) equipped with an Ultra-2 (5% phenyl-95% methylpolysiloxane bonded phase; 25 m × 0.20 mm I.D., 0.11 μm film thickness) cross-linked capillary column (Agilent Technologies). Samples (1.0 μL) were injected and conducted in a split-injection mode (10:1). GC oven temperature was set initially at 140°C for 3 min and increased to 300°C at a rate of 8°C/min with a holding time of 5 min. Helium (0.5 mL/min) and argon were used as carrier and collision gases, respectively. Ionization was performed in the electron impact ionization (EI) mode at 70 eV. SRM transitions and optimized mass parameters for each AA are summarized in **Supplementary Table 1**.

### Liquid chromatography-tandem mass spectrometry

Profiling analysis of AAs and oxidized glutathione in HL-1 cells was performed without derivatization by LC-MS/MS. Briefly, deproteinization was performed by adding acetonitrile (ACN, 40 μL) and IS (^13^C_1_-phenylalanine; 25 ng) to lysed cardiomyocytes (5.0 × 10^4^) in an Eppendorf tube, followed by mixing for 1 min. After centrifugation, the supernatant was transferred to an auto vial and injected into the LC-MS/MS system as described previously (20).

LC-MS/MS was performed using a Triple Quadrupole LCMS-8050 system (Shimadzu). An Intrada Amino Acid column (50 mm × 3.0 mm, 3 µm) was used to separate AAs. The system parameters used were as follows: ionization mode, electrospray ionization (ESI) mode; nebulizing gas flow, 3.0 L/min; heating gas flow, 10.0 L/min; interface temperature, 300°C; desolvation line (DL) temperature, 250°C. The mobile phase for AAs and oxidized glutathione was applied with a gradient elution of acetonitrile (ACN)/tetrahydrofuran (THF)/25 mM ammonium formate in water/formic acid = 9/75/16/0.3 (*v*/*v*/*v*/*v*) (A) and ACN/100 mM ammonium formate in water = 20:60 (*v*/*v*) (B). SRM transitions and optimized mass parameters for each AA are summarized in **Supplementary Table 1**.

### Star pattern recognition analysis

AA levels were determined based on a calibration curve. The mean AA levels in the PM_2.5_ treated cells were normalized to the corresponding mean levels of the control. Normalized values were plotted as lines radiating from a common central point, and the far ends were joined to produce star patterns using MS Excel (32, 33).

### Transmission electron microscope

Following PM_2.5_ treatment, cells were fixed using Karnovsky’s fixative solution (Sigma-Aldrich) for 12 h at 4°C. The fixed cells were washed with 0.1 M cacodylate buffer (pH 7.4) and treated with 1% osmium tetroxide (Polysciences, Warrington, PA, USA) in a 0.1 M cacodylate buffer for post-fixation (2 h, RT) as previously reported (20). Samples were dehydrated in a series of ethanol solutions (50 to 100%), infiltrated with propylene oxide, embedded in Epon Mixture (Polysciences), and sequentially incubated at 60°C (24 h). The sample blocks were sectioned using an ultramicrotome (Reichert-Jung, Bayreuth, Germany). For contrast staining, sections were stained with 2% uranyl acetate (Electron Microscopy Sciences, Hatfield, PA, USA) for 10 min and lead citrate (Thermo Fisher Scientific) for 5 min. Images were acquired using a transmission electron microscope (SIGMA500; Carl Zeiss, Oberkochen, Germany).

### Mitochondrial membrane potential assay

To assess mitochondrial membrane potential, 0, 10, and 100 μg/mL PM_2.5_-treated cells were incubated with 100 nM tetramethylrhodamine ethyl ester perchlorate (TMRE; Sigma-Aldrich) for 20 min at 37°C. After rinsing each well with 0.2% BSA in PBS, the red fluorescence intensity of live cells was measured at an excitation wavelength/emission wavelength of 549/575 nm using a multi-mode microplate reader (SpectraMax^®^ iD3), and fluorescence images were acquired using a fluorescence microscope (Axiovert 200M; Carl Zeiss) at the 3D immune system imaging core facility of Ajou University.

### Calcium fluctuation detection

To analyze changes in calcium fluctuations in PM_2.5_-treated HL-1 cells, following PM_2.5_-treatment (0, 10, and 100 μg/mL) for 12 h, cells were incubated with 5 μM Fluo-4 AM (green-fluorescent calcium indicator; Invitrogen) in Hepes Buffered Tyrode’s Solution for 1 h at 37°C. After rinsing each well with Tyrode’s solution, calcium fluctuation signals were detected using a fluorescence microscope (Axiovert 200M). The calcium fluctuation videos were randomly obtained six times for each condition.

### RNA extraction and quantitative real-time PCR

Total RNA was isolated from cells treated with PM_2.5_ (0, 10, and 100 μg/mL) for 12 h using Direct-zol RNA Miniprep (Zymo Research, Irvine, CA, USA), and a cDNA library from isolated RNA was synthesized using the iScript Advanced cDNA Synthesis Kit (Bio-Rad, Hercules, CA, USA) and a Thermocycler (T3000; Biometra, Jena, Germany). The transcriptomic network-related gene expression levels were measured by qRT-PCR using the SsoAdvanced™ Universal SYBR^®^ Green Supermix real-time PCR kit (Bio-Rad) using a Rotor Gene-Q system (Qiagen). Relative quantification of gene expression was calculated using the 2^-ΔΔCt^ method. The primer information is summarized in **Supplementary Table 2**.

### Statistical analysis

One-way analysis of variance (ANOVA) and Bonferroni’s *post-hoc* test were used for data analysis using the IBM SPSS Statistics 20 software (IBM Corporation, Armonk, NY, USA). *p-value < 0.05, *vs.* control; ^#^ *p*-value < 0.05, *vs.* 10 μg/mL PM_2.5_-treated cells were considered statistically significant.

## Results

### Viability and intracellular ATP and ROS levels in PM_2.5_-treated HL-1 cells

**Figure 1:**
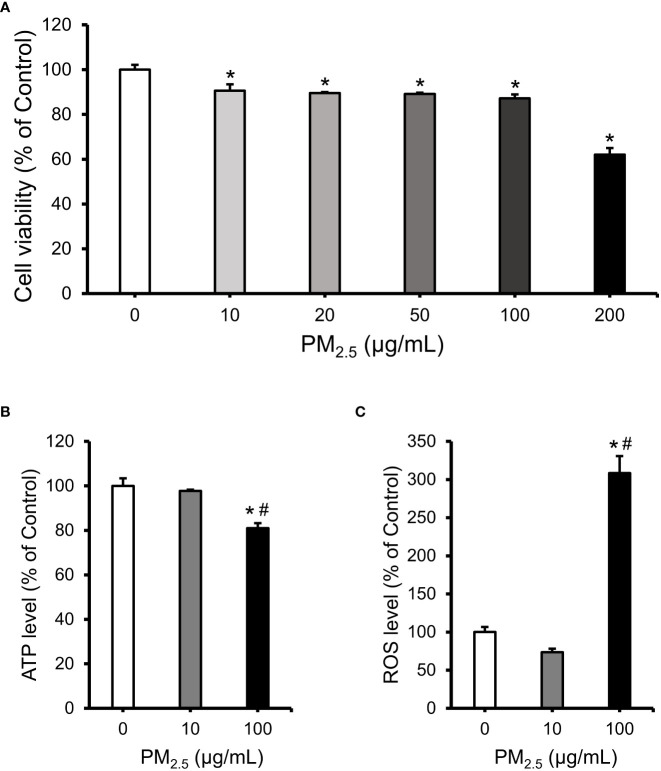
Evaluation of cell viability, intracellular ATP concentration, and ROS level in PM_2.5_-treated HL-1 cells. **(A)** Cell viability of HL-1 cells treated with PM_2.5_ (0, 10, 20, 50, 100, and 200 μg/mL) for 12 h. **(B)** The relative intracellular ATP levels in PM_2.5_-treated cells. **(C)** The relative intracellular ROS level in PM_2.5_-treated cells. Each data is shown from independently performed triplicate experiments. *p-value < 0.05 *vs.* control; ^#^ *p*-value < 0.05 *vs.* 10 μg/mL PM_2.5_-treated cells.

To assess the cytotoxicity of PM_2.5_, we analyzed the viability of 0–200 μg/mL PM_2.5_-treated HL-1 cells for 12 h. Viability was retained at approximately 90% in cells treated with up to 100 μg/mL of PM_2.5_; however, it was decreased to ~60% in those treated with 200 μg/mL of PM_2.5_ (**Figure 1A**). Therefore, we set a low dose of 10 μg/mL and a high dose of 100 μg/mL for further experiments. Intracellular ATP concentration and ROS levels showed a significant change in cells treated with 100 μg/mL PM_2.5_ compared to untreated and 10 μg/mL PM_2.5_-treated cells (**Figures 1B, C**). In cells treated with 100 μg/mL PM_2.5_, the ATP concentration decreased by approximately 20% (**Figure 1B**), and ROS accumulated over three times more than in controls (**Figure 1C**). These results suggest that a high dose of PM_2.5_, even though it might not induce cell death, may affect biological functions such as energy and redox homeostasis.

### Transcriptomic analysis of PM_2.5_-treated cardiomyocytes

**Figure 2:**
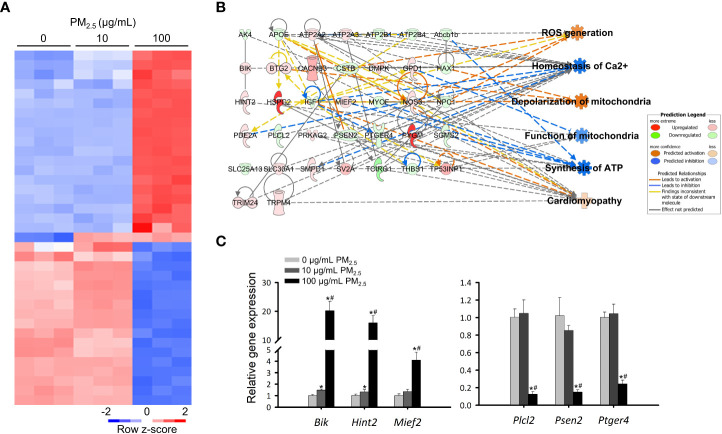
Transcriptomics analysis of PM_2.5_-treated HL-1 cells. **(A)** Heatmap of differentially expressed genes in 0, 10, and 100 μg/mL PM_2.5_-treated cells. Gene expression levels were analyzed three times per condition. **(B)** Transcriptome network with an *in silico* prediction of PM_2.5_-treated cells. A network of cellular functions and disease-related genes was generated using IPA (https://www.ingenuity.com). Green and red indicate downregulated and upregulated genes, respectively. Dotted and solid lines indicate indirect and direct relationships, respectively. Details for shape are provided in **Supplementary Figure 1**. **(C)** qPCR analysis for *Bik*, *Hint2*, *Mief2*, *Plcl2*, *Psen2*, and *Ptger4* in 0, 10, and 100 μg/mL PM_2.5_-treated cells. *Gapdh* was used as a reference gene. Data represent independently performed triplicate experiments. *p-value < 0.05 *vs.* control; ^#^ *p*-value < 0.05 *vs.* 10 μg/mL PM_2.5_-treated cells.

To investigate detailed biological changes, we analyzed the transcriptome of HL-1 cells using RNA sequencing after treatment with 0, 10, and 100 μg/mL PM_2.5_ for 12 h. We identified a total of 45,777 genes and found 6,005 genes to be differentially expressed (fold change cut-off of ± 4). We then analyzed the differentially expressed genes related to canonical pathways, diseases, and biological functions (**Supplementary Tables 3** and **4**), as well as the most significantly changed gene cluster among them (**Figure 2A**). When comparing the 10 μg/mL PM_2.5_-treated HL-1 cells to the control cells, two differentially expressed genes were found. We also identified a total of 37 differentially expressed genes in the gene cluster of the 100 μg/mL PM_2.5_-treated HL-1 cells. Next, we analyzed the transcriptomic network of the 37 genes using IPA in the 10 and 100 μg/mL PM_2.5_-treated HL-1 cells. Our analysis showed that these genes were related to six biological functions: ROS generation, homeostasis of Ca^2+^, depolarization of mitochondria, function of mitochondria, synthesis of ATP, and cardiomyopathy (**Supplementary Table 5**, **Supplementary Figures 1** and **2**). The transcriptomic network prediction revealed upregulation of ROS generation, depolarization of mitochondria, and cardiomyopathy with a downregulation in the homeostasis of Ca^2+^, function of mitochondria, and synthesis of ATP (**Figure 2B**). Among the genes in the network, the expression of Bcl2-interacting killer (*Bik*), histidine triad nucleotide binding protein 2 (*Hint2*), mitochondrial elongation factor 2 (*Mief2*), phospholipase C-like 2 (*Plcl2*), presenilin 2 (*Psen2*), and prostaglandin E receptor 4 (*Ptger4*) was validated by qPCR, and the expression trend was similar to the IPA network (**Figure 2C**).

### Metabolomic analysis of PM_2.5_-treated cardiomyocytes

**Figure 3:**
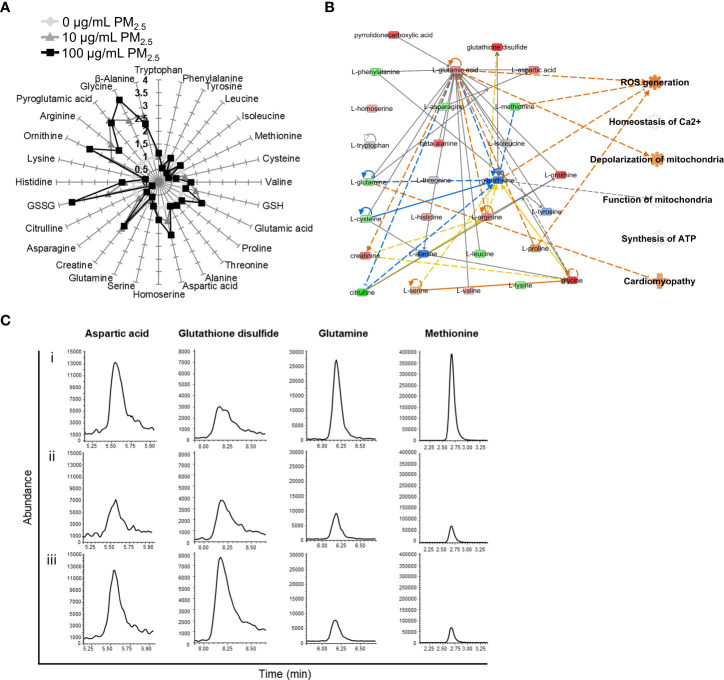
AA profiles of PM_2.5_-treated HL-1 cells. **(A)** The relative levels of 28 amino acids in 0 μg/mL PM_2.5_-treated (white), 10 μg/mL PM_2.5_-treated (gray), and 100 μg/mL PM_2.5_-treated cells (black). Each amino acid level was analyzed three times per condition. **(B)** The AA network with an *in silico* prediction of 100 μg/mL PM_2.5_-treated cells. The network was generated using IPA. Green and red indicate downregulated and upregulated amino acids, respectively. Dotted and solid lines indicate indirect and direct relationships, respectively. Details for shape and color are provided in **Supplementary Figure 1** and **Figure 2B**. **(C)** SIM chromatograms of aspartic acid, glutathione disulfide, glutamine, and methionine in **(i)** 0 μg/mL PM_2.5_-treated, **(ii)** 10 μg/mL PM_2.5_-treated, and **(iii)** 100 μg/mL PM_2.5_-treated cells.

Changes in AA profiles in 10 and 100 μg/mL PM_2.5_-treated HL-1 cells were quantified using EOC/TBDMS derivatization followed by GC-MS/MS and LC-MS/MS analyses. A total of 28 AAs were identified, of which 20 were differentially expressed (fold change cut-off of ± 1.2). In the 10 and 100 μg/mL PM_2.5_-treated HL-1 cells, 16 AAs were differentially expressed (**Figure 3A**). We analyzed the differentially expressed AAs related to canonical pathways, diseases, and biological functions (**Supplementary Tables 6** and **7**). A metabolomics network was also created using IPA, which showed the relationship between AAs and those associated with cellular functions found in the transcriptomics analysis (**Supplementary Figures 3** and **4**, **Supplementary Table 8**). Like the transcriptomics analysis, the network prediction also revealed upregulation of ROS generation, depolarization of mitochondria, and cardiomyopathy in the 10 and 100 μg/mL PM_2.5_ treated HL-1 cells (**Figure 3B**). Representative selected ion monitoring (SIM) chromatography results for the four AAs in the network, including aspartic acid, glutathione disulfide, glutamine, and methionine, are presented in **Figure 3C**.

### Metabotranscriptomic analysis of PM_2.5_-treated cardiomyocytes

**Figure 4:**
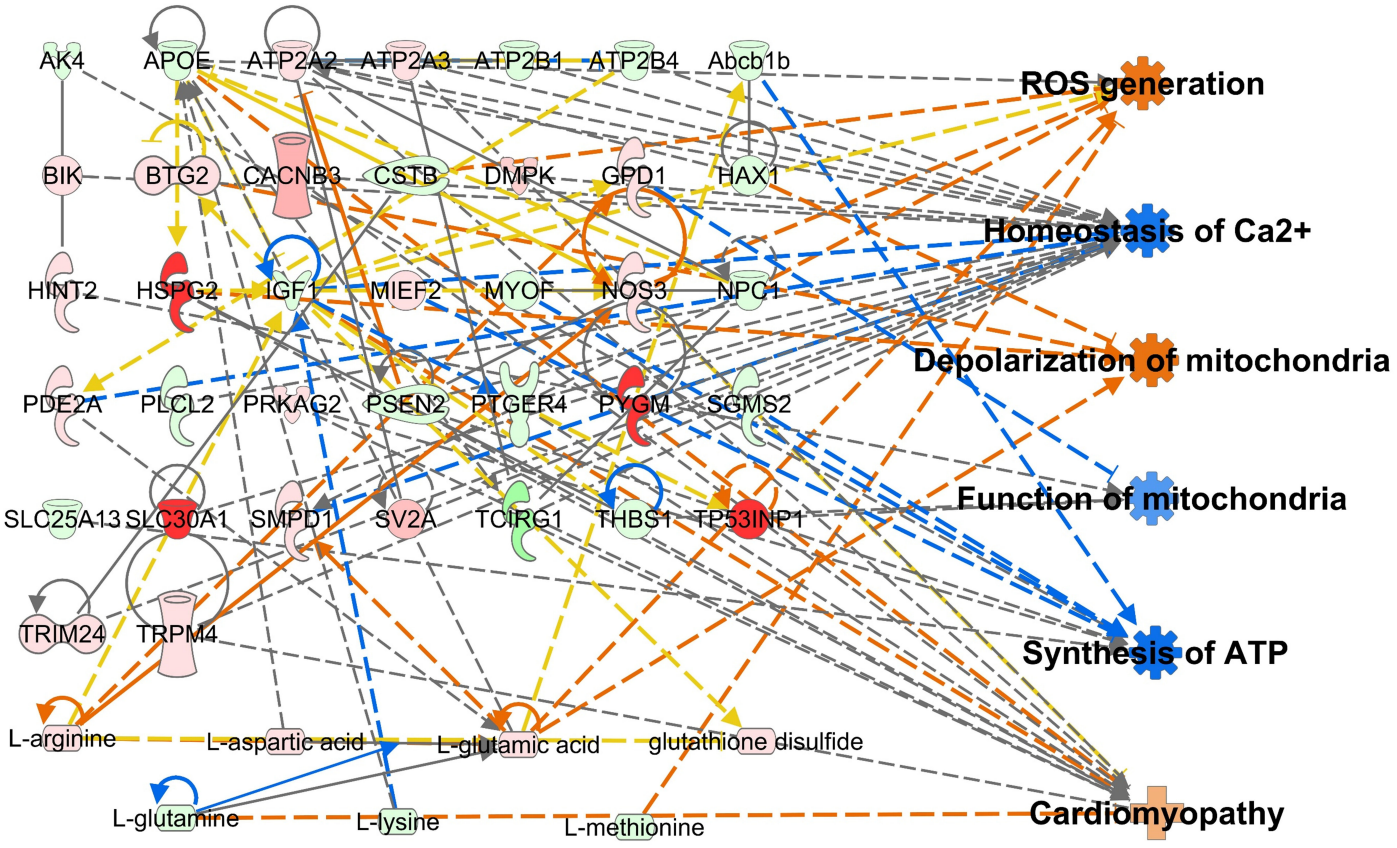
Metabotranscriptomic network with an *in silico* prediction of PM_2.5_-treated HL-1 cells. The network was generated using IPA. Green and red indicate downregulated and upregulated genes and amino acids, respectively. Dotted and solid lines indicate indirect and direct relationships, respectively. Details for shape and color are provided in **Supplementary Figure 1** and **Figure 2B**.

Integrative omics analysis was used to compensate for the limitations of single omics analysis (34–37). The transcriptome and metabolome were integrated based on biological functions, including ROS generation, homeostasis of Ca^2+^, depolarization of mitochondria, function of mitochondria, synthesis of ATP, and cardiomyopathy. Genes and AAs profiles were found to be interconnected and tightly related to the biological functions in the 10 and 100 μg/mL PM_2.5_ treated HL-1 cells (**Supplementary Figures 5** and **6**). The integrated transcriptomics and metabolomics network, namely the metabotranscriptomic network, is predicted to increase ROS generation, depolarization of mitochondria, and cardiomyopathy, and decrease homeostasis of Ca^2+^, function of mitochondria, and synthesis of ATP. This trend was more pronounced than in the single omics networks (**Figure 4**).

### Reduction in mitochondrial membrane potential in PM_2.5_-treated cardiomyocytes

**Figure 5:**
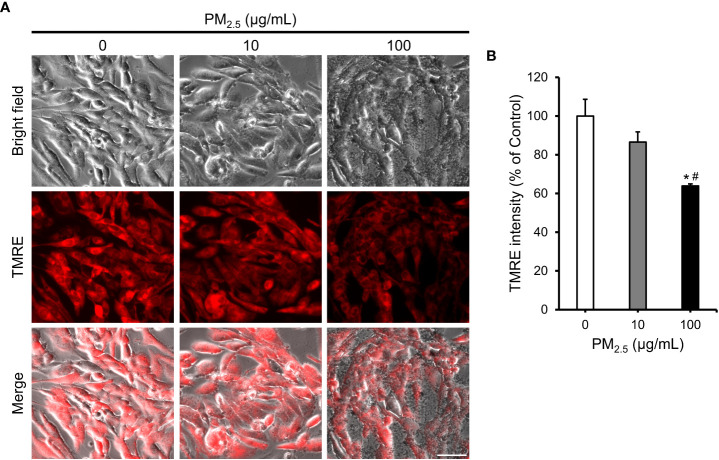
Reduction in mitochondrial membrane potential in PM_2.5_-treated HL-1 cells. **(A)** TMRE staining and bright-field images of PM_2.5_-treated cells. Scale bar = 50 μm. **(B)** Measurement of TMRE intensity in 0, 10, and 100 μg/mL PM_2.5_-treated cells. Data represent independently performed triplicate experiments. *p-value < 0.05 *vs.* control; ^#^ *p*-value < 0.05 *vs.* 10 μg/mL PM_2.5_-treated cells.

To validate the predicted biological changes, we investigated the mitochondrial membrane potential, which is highly related to homeostasis of Ca^2+^, depolarization of mitochondria, and function of mitochondria (11) in PM_2.5_-treated HL-1 cells using the fluorescence of tetramethylrhodamine ethyl ester perchlorate (TMRE) (**Figure 5A**). The TMRE intensity decreased insignificantly in 10 μg/mL PM_2.5_-treated cells (~87% that of the control); however, it decreased significantly (~64% that of the control) in the cells treated with 100 μg/mL PM_2.5_ (**Figure 5B**). This result suggests that a high dose of PM_2.5_ could reduce the electrochemical proton gradient of mitochondria, which is a critical factor in ATP synthesis.

### Impairment in movement of PM_2.5_-treated cardiomyocytes

**Figure 6:**
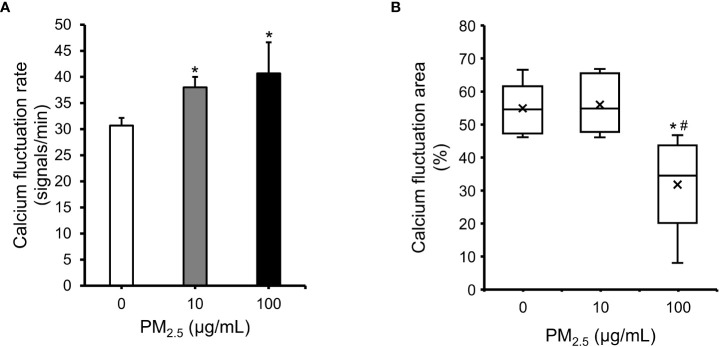
Impairment in cardiac calcium fluctuation in PM_2.5_-treated HL-1 cells. **(A)** Calcium fluctuation rates of 0, 10, and 100 μg/mL PM_2.5_-treated cells. The graph indicates the number of calcium fluctuation signals per min. **(B)** Calcium fluctuation area of PM_2.5_-treated cells. The box plot shows the cell area with calcium fluctuation signals from randomly selected microscope fields. Calcium fluctuation videos were acquired in six fields per condition. × indicates average. The internal horizontal line indicates the median value. *p-value < 0.05 *vs.* control; ^#^ *p*-value < 0.05 *vs.* 10 μg/mL PM_2.5_-treated cells.

The HL-1 cell line retained differentiated cardiac electrophysiological properties (38). Therefore, to assess the effect of PM_2.5_ on cardiac electrophysiological properties, we observed calcium fluctuation in PM_2.5_-treated HL-1 cells using a calcium indicator (**Supplementary Movies 1, 2, and 3**). Both 10 μg/mL and 100 μg/mL PM_2.5_-treated HL-1 cells showed a more rapid calcium fluctuation rate than untreated HL-1 cells (Control: 30.7 ± 1.5 signals/min, 10 μg/mL PM_2.5_: 38.0 ± 2.0 signals/min, 100 μg/mL PM_2.5_: 40.7 ± 6.0 signals/min) (**Figure 6A**). Interestingly, the calcium fluctuation area of 100 μg/mL PM_2.5_-treated HL-1 cells was significantly decreased, whereas that of 10 μg/mL PM_2.5_-treated HL-1 cells was unaffected (**Figure 6B**). Moreover, calcium fluctuation signals in some regions were rarely detected in 100 µg/mL PM_2.5_-treated HL-1 cells (only; ~8% of the total region). These results suggested that PM_2.5_ could affect the electrophysiological properties of a cardiomyocytes even in 10 μg/mL PM_2.5_-treated conditions, and especially 100 μg/mL of PM_2.5_ could impair the calcium fluctuation ability of cardiomyocytes required for heartbeat regulation.

### TEM image analysis of PM_2.5_-treated cardiomyocytes

**Figure 7:**
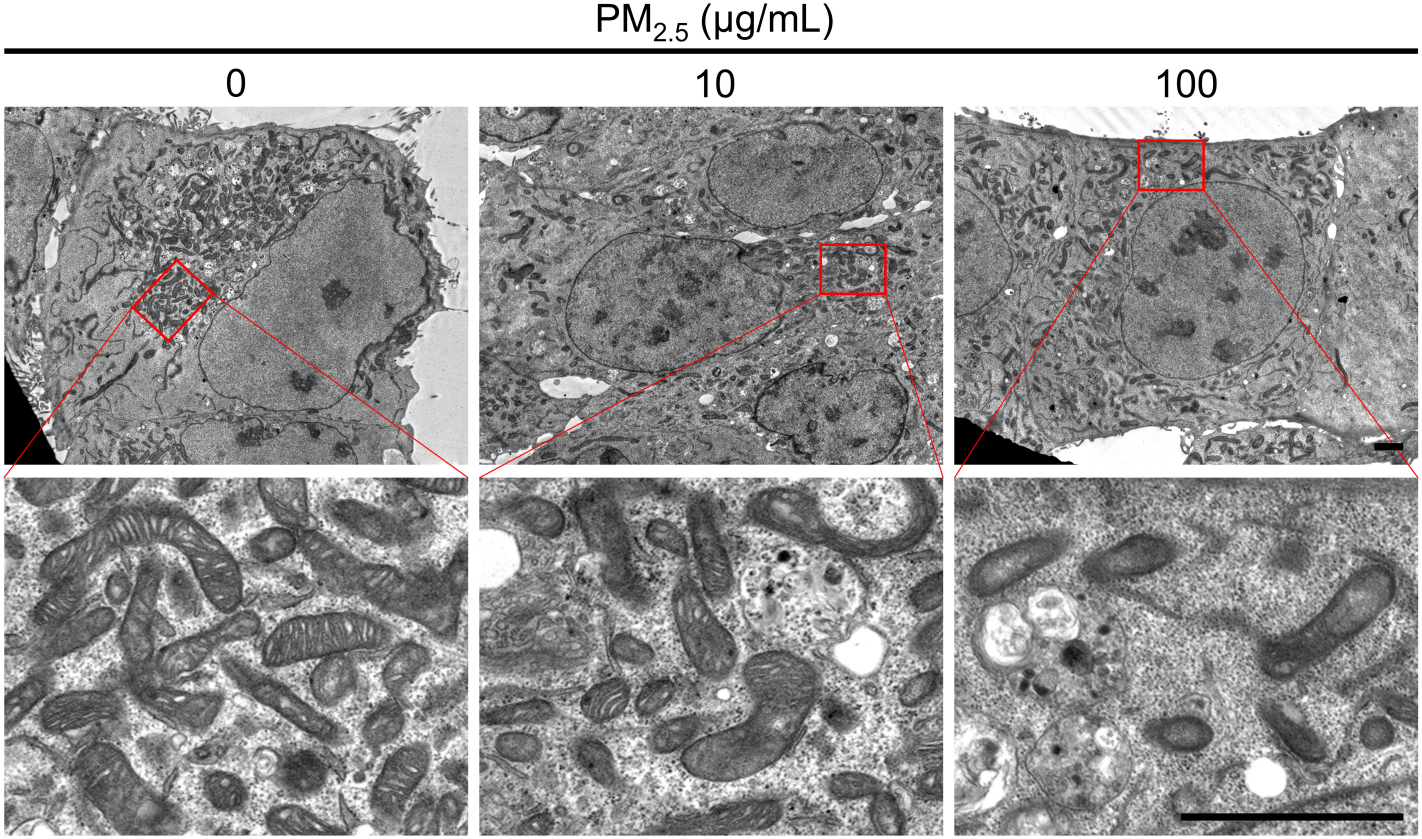
Ultrastructural analysis using TEM. TEM images represent the mitochondrial morphology of PM_2.5_-treated and non-treated HL-1 cells. Each representative image was acquired from three TEM images per condition. Magnified images are presented in each bottom panel. Scale bar = 2 μm.

To observe the mitochondrial structure of PM_2.5_-treated HL-1 cells, we captured TEM images of cells treated with 0, 10, and 100 μg/mL PM_2.5_ (**Figure 7**). In the 100 μg/mL PM_2.5_-treated cell, the structure of mitochondrial cristae was disintegrated, whereas those of non-treated cells maintained the elaborate system. In addition, cells treated with 10 and 100 μg/mL PM_2.5_ had fewer mitochondria compared to non-treated control in a dose-dependent manner. These results suggested that PM_2.5_ has an adverse effect on the mitochondria of cardiomyocytes, in particular the structure of cristae which is critical for mitochondrial functions.

## Discussion

**Figure 8:**
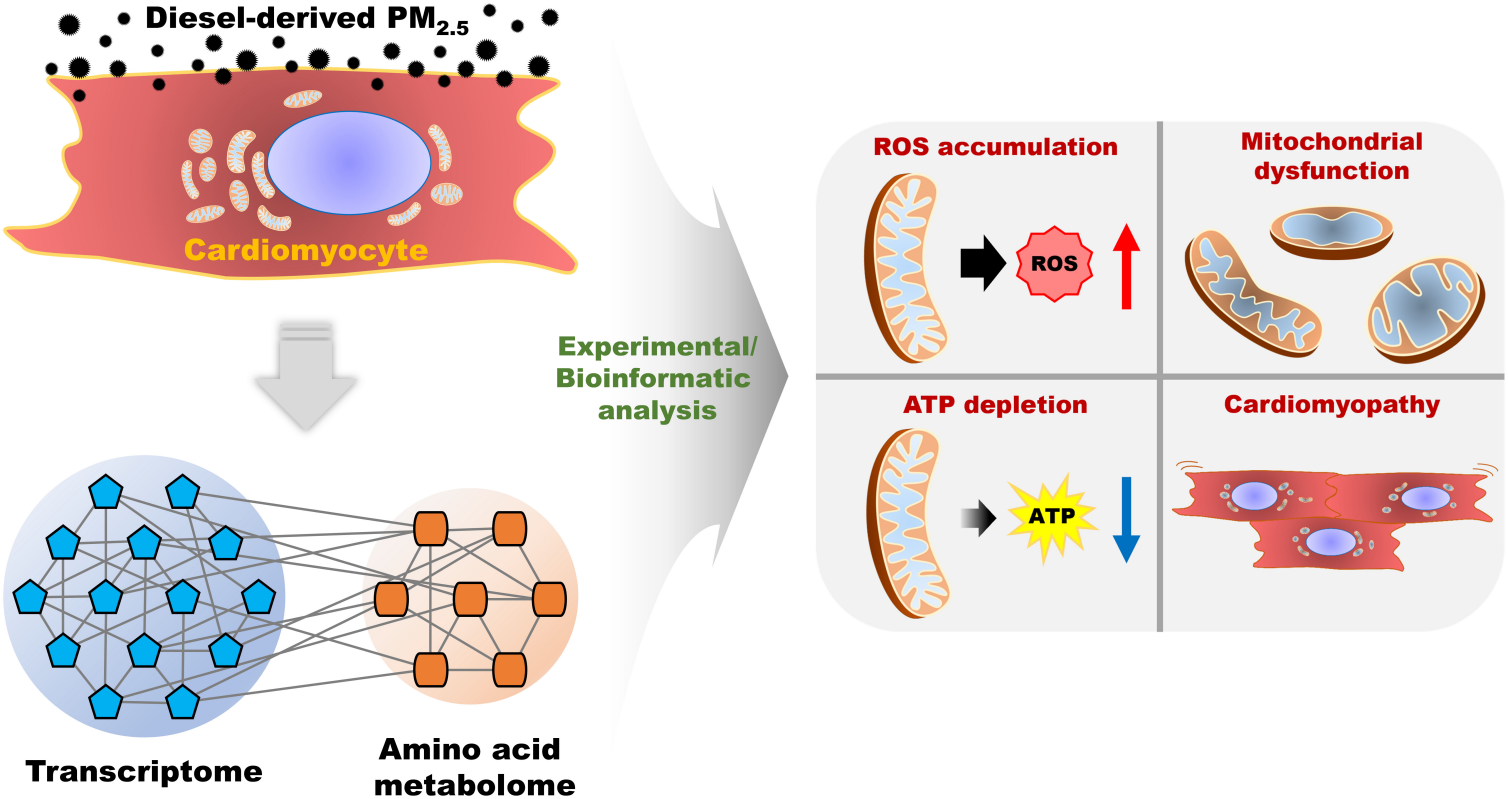
The summarized scheme of overall analysis for diesel-derived PM_2.5_ exposure in HL-1 mouse cardiomyocyte cell line.

The present study analyzed the molecular and biological changes and alterations in the transcriptome and amino acid composition in PM_2.5_-treated cardiomyocytes. Our analysis mainly focused on mitochondrial function and calcium homeostasis. We also documented the disturbances caused by PM_2.5_ in cardiomyocytes, namely its effect on cardiac movement and its relation to cardiomyopathy. We carried out the PM_2.5_-treated *in vitro* experiments and found six biological functions affected by PM_2.5_: ROS generation, homeostasis of Ca^2+^, depolarization of mitochondria, function of mitochondria, synthesis of ATP, and cardiomyopathy. Furthermore, to compensate for any experimental weakness, we integrated transcriptome and metabolome and created an integrated single omics network as in previous reports (14, 15, 20). As a result, *in silico* prediction of metabotranscriptome for six biological functions highly corresponded with the results of *in vitro* experiments. Therefore, this approach provides reliable data that reveals PM_2.5_ inducing toxicity with deleterious effects on the cardiovascular system. The overall analysis for PM_2.5_-induced biological changes is summarized in **Figure 8**. To assess the effects of diesel-derived PM_2.5_ exposure in HL-1 mouse cardiomyocyte cell lines, we performed both experimental and bioinformatic analysis in PM_2.5_-treated HL-1 cells. The PM_2.5_-induced biological changes, including ROS accumulation, mitochondrial dysfunction, ATP depletion, and cardiomyopathy, were analyzed in *in vitro* cell line and *via in silico* prediction.

The standard values for PM_2.5_ exposure set by WHO and the Chinese National Secondary Standard concentration of environmental air pollutants are 25 μg/m^3^ 24 h mean and 75 μg/m^3^ 24 h mean, respectively (39). To mimic the *in vivo* situation based on these criteria, Liu et al. reported that for the establishment of PM_2.5_ exposure mouse model (forty-eight male C57BL/6 mice, six weeks old), the 24 h mean concentration for eight weeks of exposure of control and treatment group were 23.71 and 225.05 μg/m^3^, and for 16 weeks of exposure was 21.75 and 168.00 μg/m^3^, respectively. For *in vitro* model, they treated 0, 25, 50, and 100 μg/mL of PM_2.5_ for 24 h in HL-1 cells, respectively. The biomarker expression pattern of *in vitro* and *in vivo* was found to be similar (39). Specifically, after PM_2.5_ exposure *in vivo*, the mRNA levels of an atrial natriuretic factor (ANF), brain natriuretic peptide (BNP), and β-myosin heavy chain (β-MHC) were statically upregulated by 1.28-, 0.99- and 1.16-fold, respectively. In the analysis of transcriptome after PM_2.5_ exposure in HL-1 cells, ANF, BNP, and β-MHC were significantly upregulated to 1.89-,1.65-, 1.86-fold after treatment of 50 μg/mL PM_2.5_ and increased to 2.28-, 1.99-, 2.16-fold after treatment of 100 μg/mL PM_2.5_. In addition, Sivakumar et al. reported that 100 μg/mL PM_2.5_-treated H9c2 cardiomyocytes cells showed mitochondrial dysfunction and inactivation of the PI3K/Akt signaling pathway (40). These results are consistent with our findings. Recently, this group also reported that myocardial changes in the animal model were significantly altered in 0.5 mg/mL concentration of PM_2.5_, compared to 0.05 mg/mL concentration (41). However, the pathological changes in test group with 0.05 mg/mL concentration were not statistically different from the control. Hence, they used PM_2.5_ at a concentration of 0.5 mg/mL, yielding 250 µg/m^3^ of PM_2.5_ in the exposure chamber. Based on previous reports and our preliminary data, treatment of 10 μg/mL and 100 μg/mL PM_2.5_ were established as test groups in *in vitro* experiments for extrapolating the findings to predict biological effects in *in vivo*. However, there are several limitations to analyze the clinical relevance of the concentration at which the toxic effects were observed in our study. It is difficult to assess the accurate amount of component accumulation of diesel-derived PM_2.5_ concentration in the heart due to the heterogeneous component of diesel-derived PM_2.5_ and the limitation of instrumental analysis. To validate this, there is a pressing need for large-scale *in vitro* experiments, clinical studies, and the development of high-sensitive instrumental analysis. In addition, further studies on the specific concentrations of PM_2.5_ linked with clinical relevance are warranted.

The toxicity of fine and nanoscale particles is highly dependent on the composition of the particles due to physicochemical differences of the materials and differences in toxicity of the composed chemicals (22, 42). Moreover, the composition of the PM_2.5_ varies according to PM_2.5_ sources, including region, natural circumstance, and anthropogenic factors (43). In this study, we evaluated the toxicity of diesel-derived PM_2.5_, which is certified as Standard Reference Material (SRM) by the National Institute of Standards & Technology (NIST), and the PM_2.5_ is composed with polycyclic aromatic hydrocarbons (PAHs) and nitro-substituted polycyclic aromatic hydrocarbons (Nitro-PAHs) (44, 45). PAHs and Nitro-PAHs are highly related to the toxicity of diesel-derived PM_2.5_ (46, 47). To analyze the adverse effects of PM_2.5_, it is necessary to evaluate the specific toxicity of both PAHs and Nitro-PAHs.

Zhou et al. reported that 100 or 200 μg/mL of the water-soluble fraction of PM_2.5_ or DMSO-soluble fraction of PM_2.5_ identically induced ROS and apoptosis in KGN cells, *i.e.*, ovarian granulosa cell-like human granulosa cells (48). Regardless of the solvent, chemical components of PM_2.5_ were responsible for the biological effects in the cells. Even though the cell lines are different, we assume that chemical components of PM_2.5_ are responsible for biological effects in PM_2.5_-treated HL-1 cells.

The present study showed a decrease in intracellular ATP levels and an increase in intracellular ROS levels in PM_2.5_-treated cardiomyocytes, with no significant difference in cell viability. Moreover, disruption of the mitochondrial cristae structures in PM_2.5_-treated cardiomyocytes was detected (**Figure 7**). We reported similar findings in our previous study, where human embryonic kidney 293 (HEK293) cells were treated with 50 nm silica-coated magnetic nanoparticles containing rhodamine B isothiocyanate [MNPs@SiO_2_(RITC)], which have similar characteristics to ultrafine PM (14). We believe that the generation of ROS and treatment time (12 h) were insufficient to induce cell death in both studies (14). In addition, we noted an increase in glutamate levels detected in HEK293 cells treated with MNPs@SiO_2_(RITC) (14, 49) and in cardiomyocytes treated with PM_2.5_. Thus, we concluded that the biological responses were similar for both MNPs@SiO_2_(RITC) and PM_2.5_ cells and that ROS may be a major trigger for mitochondria-related biological changes in PM_2.5_-treated cardiomyocytes.

Reduced glutathione (GSH) is the most significant non-protein thiol in mammalian cells. It acts as a scavenger of ROS through a redox reaction that yields oxidized glutathione disulfide (GSSG) (50, 51). Therefore, the GSH/GSSG ratio can be used as an indicator of oxidative stress (51, 52). In 10 μg/mL PM_2.5_-treated HL-1 cells, the level of GSSG was increased ~2-fold compared to that in controls, and the GSH level was increased ~1.5-fold. In contrast, the level of GSSG in 100 μg/mL PM_2.5_-treated HL-1 cells was increased ~3.5-fold, while that of GSH remained constant. These altered levels of GSH and GSSG suggest that the ROS-scavenging system may be impaired in PM_2.5_-treated HL-1 cells.

PM_2.5_-induced ROS disturbs calcium regulation and thus can affect cardiac movement. Cardiac movement requires calcium cycling *via* the influx and efflux of calcium in the sarcoplasmic reticulum, cytosol, and mitochondria. Any impairment of this cycle is highly related to heart failure (53). In this study, we found disturbances in the expression of ATPase sarcoplasmic/endoplasmic reticulum Ca^2+^ transporting 2/3 (*Atp2a2* and *Atp2a3*), ATPase plasma membrane Ca^2+^ transporting 1/4 (*Atp2b1* and *Atp2b4*), and voltage-gated calcium channel auxiliary subunit beta 3 (*Cacnb3*) in PM_2.5_-treated cardiomyocytes. Moreover, a decrease in intracellular ATP level is also highly related to the impairment of calcium cycling and, thus, cardiac movement (54). Imbalanced calcium homeostasis can also induce depolarization and mitochondrial dysfunction (55, 56). Thus, PM_2.5_-induced impairment of cardiac movement might be caused by disturbances in calcium regulation-related gene expression and a concomitant reduction in mitochondrial activity.

Besides the impairment of cardiac movement in 100 μg/mL PM_2.5_-treated HL-1 cells, our result showed the increment of calcium fluctuation rate in both 10 and 100 μg/mL PM_2.5_-treated cells compared to non-treated control (**Figure 6A**). This phenomenon supports previous epidemiological studies that PM_2.5_ exposure was highly related with elevated heart rate (57, 58). Since increased heart rate is considered the major cardiovascular risk factor (59), our result suggests that even relatively low concentrations of PM_2.5_ also have an adverse effect on cardiomyocytes.

Cell internalization of particles occurs through multiple pathways, including passive diffusion, phagocytosis, pinocytosis, micropinocytosis, receptor-mediated endocytosis, clathrin-mediated endocytosis, and caveolin-mediated endocytosis (60, 61). Previous studies have reported that the internalization of the fluorescence dye-labeled PM_2.5_ depends on clathrin- and caveolin-mediated endocytosis as well as other pathways according to PM_2.5_ component diversity (62). In contrast, PM emission from cells has not been well documented. Therefore, we analyzed exocytosis using integration omics in PM_2.5_-treated HL-1 cells (**Supplementary Figures 7** and **8**, **Supplementary Table 9**). We predicted that exocytosis was suppressed using the metabotranscriptomic analysis of the PM_2.5_-treated HL-1 cells (**Supplementary Figure 9**). This process is energy-dependent, and ATP levels dropped due to mitochondrial dysfunction in PM_2.5_-treated HL-1 cells. Thus, the suppressed exocytosis might correlate with the energy homeostasis of the HL-1; however, further studies using fluorescence dye labeling and super-resolution live-cell imaging are needed to elucidate the exact mechanism of exocytosis of PM_2.5_.

In addition to maintaining blood circulation, the heart also functions as an endocrine organ that produces hormones, such as atrial natriuretic peptide (ANP), brain natriuretic peptide (BNP), growth differentiation factor (GDF)-15, and endothelin-1 (63–65). As such, abnormal hormonal regulation of the heart is closely related to diseases such as hypertension, heart failure, chronic renal failure, and septic shock (64, 65). We studied the synthesis and secretion of hormone-related differentially expressed genes, AAs, and their relationship *via* the metabotranscriptomic analysis of PM_2.5_-treated HL-1 cells (**Supplementary Figures 10** and **11**, **Supplementary Table 10**), and these functions were predicted to be suppressed in PM_2.5_-treated HL-1 cells (**Supplementary Figure 12**). However, our study could not accurately describe the mechanism by which PM_2.5_ affects these functions. Future studies are needed to further document the effect of PM_2.5_ on the hormonal homeostasis of cardiomyocytes.

In this study, we analyzed molecular biological phenomena by combining transcriptomics and metabolomics with AA profiling. Recent studies have been conducted using multi-omics integration analysis for a precise and detailed understanding of the biological changes under specific conditions using transcriptomics, miRNAs, proteomics, phosphoproteomics, and metabolomics (18, 20, 66). Moreover, data processing helps find strong relationships in data using machine learning algorithms for clustering and reducing the dimensionality of the data (20). Thus, future studies with additional omics analysis combined with machine learning algorithms will further improve our understanding of PM_2.5_-induced toxicity in cardiomyocytes. In conclusion, our results suggest that exposure to PM_2.5_ can induce deleterious effects on cardiac mitochondrial function and calcium homeostasis, leading to impaired cardiac movement. This further highlights the major role fossil fuel-induced air pollution plays in the pathogenesis of cardiovascular diseases.

## Data availability statement

Data have been uploaded to the Gene Expression Omnibus https://www.ncbi.nlm.nih.gov/geo/query/acc.cgi?acc=GSE211949 under accession GSE211949.

## Author contributions

TS, SK, MP, and GL conceived and designed experiments. MJ, DK, JH, DE, and CP collected information and analyzed the data. TS, SK, MJ, NG, MP and GL wrote the manuscript. All authors contributed to the article and approved the submitted version.

## Funding

This work was supported by grants from the National Research Foundation (NRF) funded by the Ministry of Science and ICT (MSIT) in Korea (2020R1C1C1008366, 2020R1A4A4079722, and 2020M3E5D9080661).

## Acknowledgments

The authors thank Bunsoon Choi and the 3D immune system imaging core facility of Ajou University for technical assistance and cell imaging analysis.

## Conflict of interest

The authors declare that the research was conducted in the absence of any commercial or financial relationships that could be construed as a potential conflict of interest.

## Publisher's note

All claims expressed in this article are solely those of the authors and do not necessarily represent those of their affiliated organizations, or those of the publisher, the editors and the reviewers. Any product that may be evaluated in this article, or claim that may be made by its manufacturer, is not guaranteed or endorsed by the publisher.
