# Supplementary material for: Diesel-derived PM2.5 induces impairment of cardiac movement followed by mitochondria dysfunction in cardiomyocytes
Source: Front Endocrinol (Lausanne). 2022 Sep 28;13:999475. doi: 10.3389/fendo.2022.999475 (PMC9554599; doi:10.3389/fendo.2022.999475)
Supplement: Supplementary file 1 [file DataSheet_1.docx]

Supplementary Table 1. SRM transitions and optimized mass parameters for 28 AAs

| **No** | **Metabolite** | **Q1** | **Q2** | **Q3** |
| --- | --- | --- | --- | --- |
|  |  | **Precursor ion (*m*/*z*)** | **CE (V)** | **Product ion (*m*/*z*)** |
| 1 | Tryptophan | 205 | -10 | 188 |
| 2 | Phenylalanine | 166 | -15 | 120 |
| 3 | Tyrosine | 182 | -27 | 91 |
| 4 | Leucine | 132 | -10 | 86 |
| 5 | Isoleucine | 132 | -10 | 86 |
| 6 | Methionine | 150 | -16 | 56 |
| 7 | Cysteine | 122 | -22 | 59 |
| 8 | Valine | 118 | -10 | 72 |
| 9 | GSH | 307 | -12 | 179 |
| 10 | Glutamic acid | 148 | -15 | 84 |
| 11 | Proline | 116 | -15 | 70 |
| 12 | Threonine | 120 | -11 | 74 |
| 13 | Alanine | 90 | -11 | 44 |
| 14 | Aspartic acid | 134 | -13 | 74 |
| 15 | Homoserine | 120 | -12 | 74 |
| 16 | Serine | 106 | -11 | 60 |
| 17 | Glutamine | 147 | -16 | 84 |
| 18 | Creatine | 132 | -13 | 90 |
| 19 | Asparagine | 133 | -15 | 74 |
| 20 | Citrulline | 176 | -22 | 70 |
| 21 | GSSG | 613 | -22 | 355 |
| 22 | Histidine | 156 | -15 | 110 |
| 23 | Lysine | 147 | -15 | 84 |
| 24 | Ornithine | 132 | -17 | 70 |
| 25 | Arginine | 175 | -25 | 70 |
| 26 | Pyroglutamic acid | 128 | 15 | 84 |
| 27 | Glycine | 204 | 5 | 176 |
| 28 | β-Alanine | 218 | 15 | 129 |

**Supplementary Table 2.** Sequences of quantitative real-time PCR primers for transcriptomic network-related genes

| **Gene Name** | **Symbol** | **NCBI Ref. seq** | **Direction** | **Primer sequence (5’-3’)** |
| --- | --- | --- | --- | --- |
| Bcl2-interacting killer | Bik | NM_007546.2 | Forward | GGGATTGCTATACACAGACTCG |
|  |  |  | Reverse | CTCTCCAGGACCAGATGTTTTC |
| Histidine triad nucleotide binding protein 2 | Hint2 | BC086940.1 | Forward | AGTGCCTTGTGTTCCGTG |
|  |  |  | Reverse | GAGTAAGTGTCCTAGAAGCTGC |
| Mitochondrial elongation factor 2 | Mief2 | NM_001009927.2 | Forward | CCAGAAGCAGAGGAAACAGAG |
|  |  |  | Reverse | TTACAGCCAGGGTAGCAATG |
| Phospholipase C-like 2 | Plcl2 | NM_013880.3 | Forward | GGAATCTTATCTACCATCCCCAG |
|  |  |  | Reverse | CATTCTCTTTCCCCATCCTCTG |
| Presenilin 2 | Psen2 | BC010403.2 | Forward | TGTCACGCTGTGTATGATCG |
|  |  |  | Reverse | TGTTAAGCACGGAGTTGAGG |
| Prostaglandin E receptor 4 | Ptger4 | BC011193.1 | Forward | CGGGAGTTAAAGGAGATCAGC |
|  |  |  | Reverse | AAAGTTCTCAGCGAGGTGG |
| Glyceraldehyde-3-phosphate dehydrogenase | Gapdh | NM_001289726.1 | Forward | GAAGACTGTGGATGGCCC |
|  |  |  | Reverse | CCATGCCAGTGAGCTTCC |

**Supplementary Table 3.** Top 20 canonical pathways algorithmically generated by ingenuity pathway analysis of the transcriptome of PM_2.5_-treated HL-1 cells

| **Canonical Pathways** | **-log(*p*-value)** | **Number of genes** |
| --- | --- | --- |
| Axonal Guidance Signaling | 6.8 | 65 |
| Cellular Effects of Sildenafil (Viagra) | 4.91 | 25 |
| Notch Signaling | 4.68 | 11 |
| Actin Cytoskeleton Signaling | 4.58 | 34 |
| Hepatic Fibrosis Signaling Pathway | 4.5 | 50 |
| Molecular Mechanisms of Cancer | 4.41 | 52 |
| Natural Killer Cell Signaling | 4.39 | 29 |
| Osteoarthritis Pathway | 4.15 | 32 |
| Cardiac Hypertrophy Signaling (Enhanced) | 4.08 | 59 |
| Caveolar-mediated Endocytosis Signaling | 4.06 | 15 |
| RAC Signaling | 4.06 | 22 |
| Glutathione-mediated Detoxification | 4.03 | 10 |
| Phagosome Formation | 3.98 | 71 |
| Protein Kinase A Signaling | 3.92 | 47 |
| TNFR2 Signaling | 3.82 | 9 |
| Neuroinflammation Signaling Pathway | 3.65 | 38 |
| Antioxidant Action of Vitamin C | 3.47 | 18 |
| Hepatic Fibrosis / Hepatic Stellate Cell Activation | 3.41 | 26 |
| Virus Entry via Endocytic Pathways | 3.4 | 17 |
| Insulin Secretion Signaling Pathway | 3.34 | 33 |

**Supplementary Table 4.** Top 20 diseases or functions annotation algorithmically generated by ingenuity pathway analysis of the transcriptome of PM_2.5_-treated HL-1 cells

| **Diseases or Functions Annotation** | ***p*-value** | **Number of genes** |
| --- | --- | --- |
| Head and neck tumor | 1.62×10^-68^ | 1348 |
| Nonpituitary endocrine tumor | 7.22×10^-66^ | 1229 |
| Thyroid gland tumor | 4.07×10^-65^ | 1212 |
| Thyroid carcinoma | 5.07×10^-65^ | 1208 |
| Neck neoplasm | 9.13×10^-65^ | 1219 |
| Head and neck cancer | 1.54×10^-64^ | 1285 |
| Tumorigenesis of tissue | 6.84×10^-64^ | 1446 |
| Endocrine carcinoma | 1.72×10^-63^ | 1242 |
| Endocrine gland tumor | 1.39×10^-62^ | 1252 |
| Epithelial neoplasm | 2.39×10^-62^ | 1441 |
| Non-melanoma solid tumor | 5.87×10^-62^ | 1451 |
| Non-hematological solid tumor | 1.70×10^-61^ | 1455 |
| Carcinoma | 8.00×10^-61^ | 1437 |
| Head and neck carcinoma | 1.17×10^-60^ | 1246 |
| Nonhematologic malignant neoplasm | 6.22×10^-60^ | 1449 |
| Cancer of secretory structure | 1.07×10^-57^ | 1277 |
| Solid tumor | 1.88×10^-55^ | 1464 |
| Malignant solid tumor | 9.96×10^-55^ | 1458 |
| Extracranial solid tumor | 1.40×10^-54^ | 1455 |
| Cancer | 6.42×10^-54^ | 1461 |

**Supplementary Table 5.** Ingenuity pathway analysis-based transcriptome profiles of HL-1 cells treated with PM_2.5_

| Entrez gene name | Symbol | Entrez Gene ID | Location | Signal fold change^a^ | |
| --- | --- | --- | --- | --- | --- |
|  |  |  |  | 10 μg/mL | 100 μg/mL |
| ATP-binding cassette, sub-family B (MDR/TAP), member 1B | Abcb1b | 18669 | Plasma Membrane | -1.32 | -4.57 |
| adenylate kinase 4 | AK4 | 11639 | Cytoplasm | 1.23 | -16.08 |
| apolipoprotein E | APOE | 11816 | Extracellular Space | -1.41 | -4.88 |
| ATPase sarcoplasmic/endoplasmic reticulum Ca2+ transporting 2 | ATP2A2 | 11938 | Cytoplasm | -1.12 | 4.12 |
| ATPase sarcoplasmic/endoplasmic reticulum Ca2+ transporting 3 | ATP2A3 | 53313 | Cytoplasm | 2.11 | 22.87 |
| ATPase plasma membrane Ca2+ transporting 1 | ATP2B1 | 67972 | Plasma Membrane | 1.21 | -5.53 |
| ATPase plasma membrane Ca2+ transporting 4 | ATP2B4 | 381290 | Plasma Membrane | -1.04 | -4.92 |
| BCL2 interacting killer | BIK | 12124 | Cytoplasm | -1.30 | 17.25 |
| BTG anti-proliferation factor 2 | BTG2 | 12227 | Nucleus | 1.37 | 17.93 |
| calcium voltage-gated channel auxiliary subunit beta 3 | CACNB3 | 12297 | Plasma Membrane | 1.25 | 87.28 |
| DM1 protein kinase | DMPK | 13400 | Cytoplasm | -1.79 | 53.78 |
| glycerol-3-phosphate dehydrogenase 1 | GPD1 | 14555 | Cytoplasm | -1.42 | 14.63 |
| HCLS1 associated protein X-1 | HAX1 | 23897 | Cytoplasm | 1.30 | -4.69 |
| histidine triad nucleotide binding protein 2 | HINT2 | 68917 | Cytoplasm | -1.28 | 12.35 |
| heparan sulfate proteoglycan 2 | HSPG2 | 15530 | Extracellular Space | 1.04 | 2779.21 |
| insulin like growth factor 1 | IGF1 | 16000 | Extracellular Space | -1.19 | -25.31 |
| mitochondrial elongation factor 2 | MIEF2 | 237781 | Cytoplasm | 1.55 | 4.82 |
| myoferlin | MYOF | 226101 | Nucleus | 1.23 | -14.96 |
| phosphodiesterase 2A | PDE2A | 207728 | Cytoplasm | -1.05 | 10.00 |
| phospholipase C like 2 | PLCL2 | 224860 | Cytoplasm | 1.01 | -4.20 |
| protein kinase AMP-activated non-catalytic subunit gamma 2 | PRKAG2 | 108099 | Cytoplasm | -1.03 | 4.38 |
| presenilin 2 | PSEN2 | 19165 | Cytoplasm | -1.16 | -5.54 |
| prostaglandin E receptor 4 | PTGER4 | 19219 | Plasma Membrane | -1.32 | -7.58 |
| glycogen phosphorylase, muscle associated | PYGM | 19309 | Cytoplasm | 1.24 | 545.23 |
| sphingomyelin synthase 2 | SGMS2 | 74442 | Plasma Membrane | 1.65 | -18.59 |
| solute carrier family 25 member 13 | SLC25A13 | 50799 | Cytoplasm | -1.21 | -10.19 |
| solute carrier family 30 member 1 | SLC30A1 | 22782 | Plasma Membrane | 4.13 | 4.22 |
| sphingomyelin phosphodiesterase 1 | SMPD1 | 20597 | Cytoplasm | 1.14 | 4.83 |
| synaptic vesicle glycoprotein 2A | SV2A | 64051 | Cytoplasm | -1.28 | 71.41 |
| T cell immune regulator 1, ATPase H+ transporting V0 subunit a3 | TCIRG1 | 27060 | Plasma Membrane | -1.01 | -193.73 |
| thrombospondin 1 | THBS1 | 21825 | Extracellular Space | 1.99 | -4.30 |
| tumor protein p53 inducible nuclear protein 1 | TP53INP1 | 60599 | Nucleus | 6.48 | 86.69 |
| tripartite motif containing 24 | TRIM24 | 21848 | Nucleus | -1.05 | 9.94 |
| transient receptor potential cation channel subfamily M member 4 | TRPM4 | 68667 | Plasma Membrane | 1.08 | 9.32 |

^a^Normalized signal fold change of signal in treated groups with PM_2.5_ to corresponding signal of control group

**Supplementary Table 6.** Top 20 canonical pathways algorithmically generated by ingenuity pathway analysis of amino acids in PM_2.5_-treated HL-1 cells

| **Canonical Pathways** | **-log(*p*-value)** | **Number of amino acids** |
| --- | --- | --- |
| tRNA Charging | 28.5 | 13 |
| Superpathway of Citrulline Metabolism | 12.7 | 6 |
| Arginine Biosynthesis IV | 11.3 | 5 |
| Citrulline Biosynthesis | 11 | 5 |
| Asparagine Biosynthesis I | 10.8 | 4 |
| Proline Biosynthesis II (from Arginine) | 9.19 | 4 |
| Urea Cycle | 8.99 | 4 |
| γ-glutamyl Cycle | 8.5 | 4 |
| Phenylalanine Degradation IV (Mammalian, via Side Chain) | 8.01 | 4 |
| Purine Nucleotides De Novo Biosynthesis II | 7.68 | 4 |
| Glycine Degradation (Creatine Biosynthesis) | 7.54 | 3 |
| Glutathione Biosynthesis | 7.25 | 3 |
| Arginine Degradation I (Arginase Pathway) | 7.01 | 3 |
| Arginine Degradation VI (Arginase 2 Pathway) | 6.72 | 3 |
| Citrulline-Nitric Oxide Cycle | 6.72 | 3 |
| 5-aminoimidazole Ribonucleotide Biosynthesis I | 6.56 | 3 |
| Uridine-5'-phosphate Biosynthesis | 6.41 | 3 |
| Asparagine Degradation I | 5.29 | 2 |
| Glutamine Degradation I | 5.29 | 2 |
| Ferroptosis Signaling Pathway | 5.26 | 4 |

**Supplementary Table 7.** Top 20 diseases or functions annotation algorithmically generated by ingenuity pathway analysis of amino acids in PM_2.5_-treated HL-1 cells

| **Diseases or Functions Annotation** | ***p*-value** | **Number of amino acids** |
| --- | --- | --- |
| Transport of amino acids | 4.78×10^-20^ | 12 |
| Mucopolysaccharidosis type I | 1.90×10^-17^ | 8 |
| Uptake of amino acids | 1.03×10^-15^ | 9 |
| Growth of bacteria | 2.09×10^-15^ | 9 |
| Uptake of L-amino acid | 2.62×10^-15^ | 8 |
| Uptake of L-alanine | 3.63×10^-15^ | 6 |
| Lysosomal storage disease | 1.11×10^-14^ | 9 |
| Growth of Yersinia pestis | 1.08×10^-13^ | 6 |
| Efflux of neutral amino acid | 2.47×10^-13^ | 5 |
| Efflux of L-amino acid | 1.20×10^-11^ | 5 |
| Entry into S phase of hepatocytes | 1.48×10^-11^ | 4 |
| Peroxidation of lipid | 2.19×10^-11^ | 7 |
| Arrest in cell cycle progression of hepatocytes | 4.44×10^-11^ | 4 |
| Efflux of L-alanine | 1.51×10^-10^ | 4 |
| Synthesis of protein | 1.36×10^-9^ | 9 |
| Concentration of glutathione | 1.92×10^-9^ | 6 |
| Quantity of amino acids | 2.46×10^-9^ | 7 |
| Activation of brain | 4.15×10^-9^ | 5 |
| Transport of molecule | 4.75×10^-9^ | 13 |
| Quantity of nitric oxide | 1.09×10^-8^ | 5 |

**Supplementary Table 8.** Ingenuity pathway analysis-based metabolomic profiles of HL-1 cells treated with PM_2.5_

| Symbol | ID^b^ | Location | Signal fold change^a^ | |
| --- | --- | --- | --- | --- |
|  |  |  | 10 μg/mL | 100 μg/mL |
| glutathione disulfide | 27025-41-8 | Other | 2.03 | 3.49 |
| L-arginine | 74-79-3 | Other | 2.39 | 2.00 |
| L-aspartic acid | 56-84-8 | Other | 1.55 | 2.12 |
| L-glutamic acid | 56-86-0 | Other | 1.83 | 1.86 |
| L-glutamine | 56-85-9 | Other | -1.28 | -1.61 |
| L-lysine | 56-87-1 | Other | -1.86 | -1.83 |
| L-methionine | 63-68-3 | Other | -1.91 | -2.46 |

^a^Fold change of normalized signal in PM_2.5_ treated group relative to corresponding control group. ^b^CAS Registry Number for identifying the AAs.

**Supplementary Table 9.** Ingenuity pathway analysis-based profiles of transcriptome and amino acids of HL-1 cells treated with PM_2.5_ in a network of exocytosis function

| Entrez gene name | Symbol | CAS Registry Number/Entrez Gene ID^b^ | Location | Signal fold change^a^ | |
| --- | --- | --- | --- | --- | --- |
|  |  |  |  | 10 μg/mL | 100 μg/mL |
| amyloid beta precursor protein binding family A member 1 | APBA1 | 319924 | Cytoplasm | 1.45 | 5.46 |
| ATPase sarcoplasmic/endoplasmic reticulum Ca2+ transporting 2 | ATP2A2 | 11938 | Cytoplasm | -1.12 | 4.12 |
| calcium voltage-gated channel subunit alpha1 A | CACNA1A | 12286 | Plasma Membrane | 1.15 | -5.86 |
| calcium dependent secretion activator | CADPS | 27062 | Plasma Membrane | -1.17 | -10.15 |
| dynamin 1 | DNM1 | 13429 | Cytoplasm | 1.44 | -6.01 |
| exocyst complex component 3 like 1 | EXOC3L1 | 277978 | Cytoplasm | 1.44 | 8.10 |
| Fc fragment of IgG receptor IIIa | FCGR3A/FCGR3B | 246256 | Plasma Membrane | 1.57 | -106.62 |
| GEM interacting protein | GMIP | 78816 | Cytoplasm | -1.30 | -8.52 |
| insulin like growth factor 1 | IGF1 | 16000 | Extracellular Space | -1.19 | -25.31 |
| potassium voltage-gated channel subfamily B member 1 | KCNB1 | 16500 | Plasma Membrane | -1.51 | 10.27 |
| KIT ligand | KITLG | 17311 | Extracellular Space | 1.57 | -9.29 |
| NSF attachment protein beta | NAPB | 17957 | Cytoplasm | 1.40 | 10.74 |
| nitric oxide synthase 3 | NOS3 | 18127 | Cytoplasm | 1.02 | 5.57 |
| prion protein | PRNP | 19122 | Plasma Membrane | 1.50 | 8.55 |
| Rap guanine nucleotide exchange factor 4 | RAPGEF4 | 56508 | Cytoplasm | -1.07 | 29.92 |
| secretory carrier membrane protein 5 | SCAMP5 | 56807 | Cytoplasm | -1.00 | -7.10 |
| spectrin beta, non-erythrocytic 2 | SPTBN2 | 20743 | Cytoplasm | 1.30 | -5.73 |
| SRC kinase signaling inhibitor 1 | SRCIN1 | 56013 | Cytoplasm | 1.52 | 6.68 |
| syntaxin 1A | STX1A | 20907 | Cytoplasm | 1.10 | -26.41 |
| syntaxin 3 | STX3 | 20908 | Plasma Membrane | 1.76 | -43.97 |
| t-complex 11 | TCP11 | 21463 | Cytoplasm | 1.20 | 7.53 |
| toll like receptor 2 | TLR2 | 24088 | Plasma Membrane | -1.16 | -25.13 |
| unc-13 homolog A | UNC13A | 382018 | Plasma Membrane | -1.23 | -17.22 |
|  | L-glutamic acid | 56-86-0 | Other | 1.83 | 1.86 |
|  | glycine | 56-40-6 | Other | 2.69 | 3.58 |

^a^Normalized signal fold change of signal in treated groups with PM_2.5_ to corresponding signal of control group ^b^Entrez Gene ID and CAS Registry Number for identifying the genes and amino acids, respectively

**Supplementary Table 10.** Ingenuity pathway analysis-based profiles of transcriptome and amino acids of HL-1 cells treated with PM_2.5_ in a network of synthesis and secretion of hormone functions

| Entrez gene name | Symbol | CAS Registry Number/Entrez Gene ID^b^ | Location | Signal fold change^a^ | |
| --- | --- | --- | --- | --- | --- |
|  |  |  |  | 10 μg/mL | 100 μg/mL |
| ATP binding cassette subfamily G member 1 | ABCG1 | 11307 | Plasma Membrane | -1.03 | -36.19 |
| actin alpha 2, smooth muscle | ACTA2 | 11475 | Cytoplasm | 1.19 | 808.56 |
| annexin A1 | ANXA1 | 16952 | Plasma Membrane | 1.29 | -22.69 |
| activating transcription factor 3 | ATF3 | 11910 | Nucleus | 1.44 | -16.17 |
| complement C3 | C3 | 12266 | Extracellular Space | -1.34 | -142.44 |
| calcium voltage-gated channel subunit alpha1 A | CACNA1A | 12286 | Plasma Membrane | 1.15 | -5.86 |
| calcium dependent secretion activator | CADPS | 27062 | Plasma Membrane | -1.17 | -10.15 |
| cathepsin B | CTSB | 13030 | Cytoplasm | 1.28 | -15.64 |
| cathepsin K | CTSK | 13038 | Cytoplasm | 1.26 | -1951.09 |
| cathepsin V | CTSV | 13039 | Cytoplasm | 1.05 | -8.77 |
| diacylglycerol lipase alpha | DAGLA | 269060 | Plasma Membrane | 1.53 | 6.71 |
| ETS variant transcription factor 1 | ETV1 | 14009 | Nucleus | -1.32 | -20.34 |
| exocyst complex component 3 like 1 | EXOC3L1 | 277978 | Cytoplasm | 1.44 | 8.10 |
| frizzled class receptor 4 | FZD4 | 14366 | Plasma Membrane | 1.13 | 172.55 |
| G protein subunit alpha 11 | GNA11 | 14672 | Plasma Membrane | -1.11 | 6.70 |
| homeostatic iron regulator | HFE | 15216 | Plasma Membrane | -1.21 | -5.71 |
| histidine triad nucleotide binding protein 2 | HINT2 | 68917 | Cytoplasm | -1.28 | 12.35 |
| 3-hydroxy-3-methylglutaryl-CoA reductase | HMGCR | 15357 | Cytoplasm | 1.24 | -8.08 |
| H6 family homeobox 2 | HMX2 | 15372 | Nucleus | -1.20 | 8.18 |
| insulin like growth factor 1 | IGF1 | 16000 | Extracellular Space | -1.19 | -25.31 |
| KISS1 receptor | KISS1R | 114229 | Plasma Membrane | 1.74 | 63.53 |
| KIT ligand | KITLG | 17311 | Extracellular Space | 1.57 | -9.29 |
| latent transforming growth factor beta binding protein 4 | LTBP4 | 108075 | Extracellular Space | -1.03 | 6.76 |
| MAF bZIP transcription factor A | MAFA | 378435 | Nucleus | 1.49 | 30.91 |
| mastermind-like domain containing 1 | Mamld1 | 333639 | Nucleus | 1.58 | 116.55 |
| matrix metallopeptidase 9 | MMP9 | 17395 | Extracellular Space | -1.38 | -42.86 |
| metastasis associated 1 family member 3 | MTA3 | 116871 | Nucleus | 1.21 | -424.03 |
| neuromedin B | NMB | 68039 | Extracellular Space | -1.42 | 6.96 |
| natriuretic peptide receptor 1 | NPR1 | 18160 | Plasma Membrane | -1.11 | 5.55 |
| nuclear receptor subfamily 4 group A member 1 | NR4A1 | 15370 | Nucleus | -1.36 | 4.40 |
| platelet derived growth factor subunit B | PDGFB | 18591 | Extracellular Space | 1.41 | -10.85 |
| Rap guanine nucleotide exchange factor 4 | RAPGEF4 | 56508 | Cytoplasm | -1.07 | 29.92 |
| RUNX family transcription factor 2 | RUNX2 | 12393 | Nucleus | -1.01 | -23.80 |
| SET nuclear proto-oncogene | SET | 56086 | Nucleus | -1.17 | -5.02 |
| syntaxin 1A | STX1A | 20907 | Cytoplasm | 1.10 | -26.41 |
| thromboxane A synthase 1 | TBXAS1 | 21391 | Plasma Membrane | 1.20 | -10.80 |
| transforming growth factor beta 1 | TGFB1 | 21803 | Extracellular Space | -1.25 | -9.53 |
| transcriptional regulating factor 1 | TRERF1 | 224829 | Nucleus | -1.18 | -205.91 |
| urocortin 2 | UCN2 | 171530 | Extracellular Space | -1.36 | -12.96 |
| WW domain containing transcription regulator 1 | WWTR1 | 97064 | Nucleus | -1.00 | 7.92 |
|  | L-arginine | 74-79-3 | Other | 2.39 | 2.00 |

^a^Normalized signal fold change of signal in treated groups with PM_2.5_ to corresponding signal of control group ^b^Entrez Gene ID and CAS Registry Number for identifying the genes and amino acids, respectively


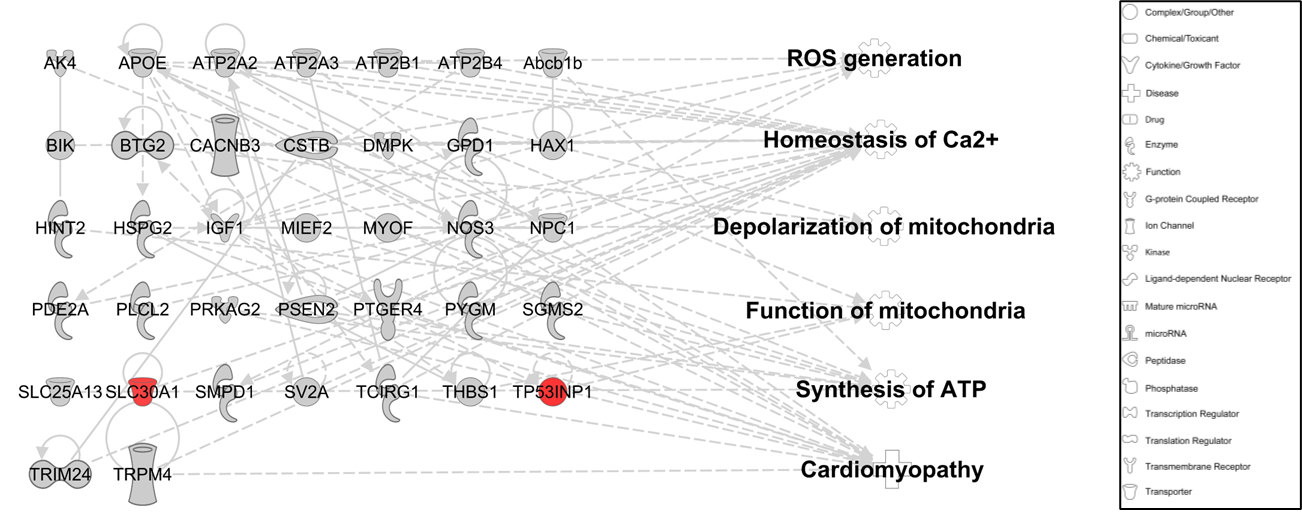


**Supplementary Figure 1 |** Functional analysis of the transcriptomic network of 10 µg/mL PM_2.5_-treated HL-1 cells using IPA. A fold change of ± 4 was used as the cut-off value. Red and green areas indicate genes whose expressions were upregulated and downregulated, respectively. Path designer shapes originated from ingenuity systems (http://www.ingenuity.com).

**
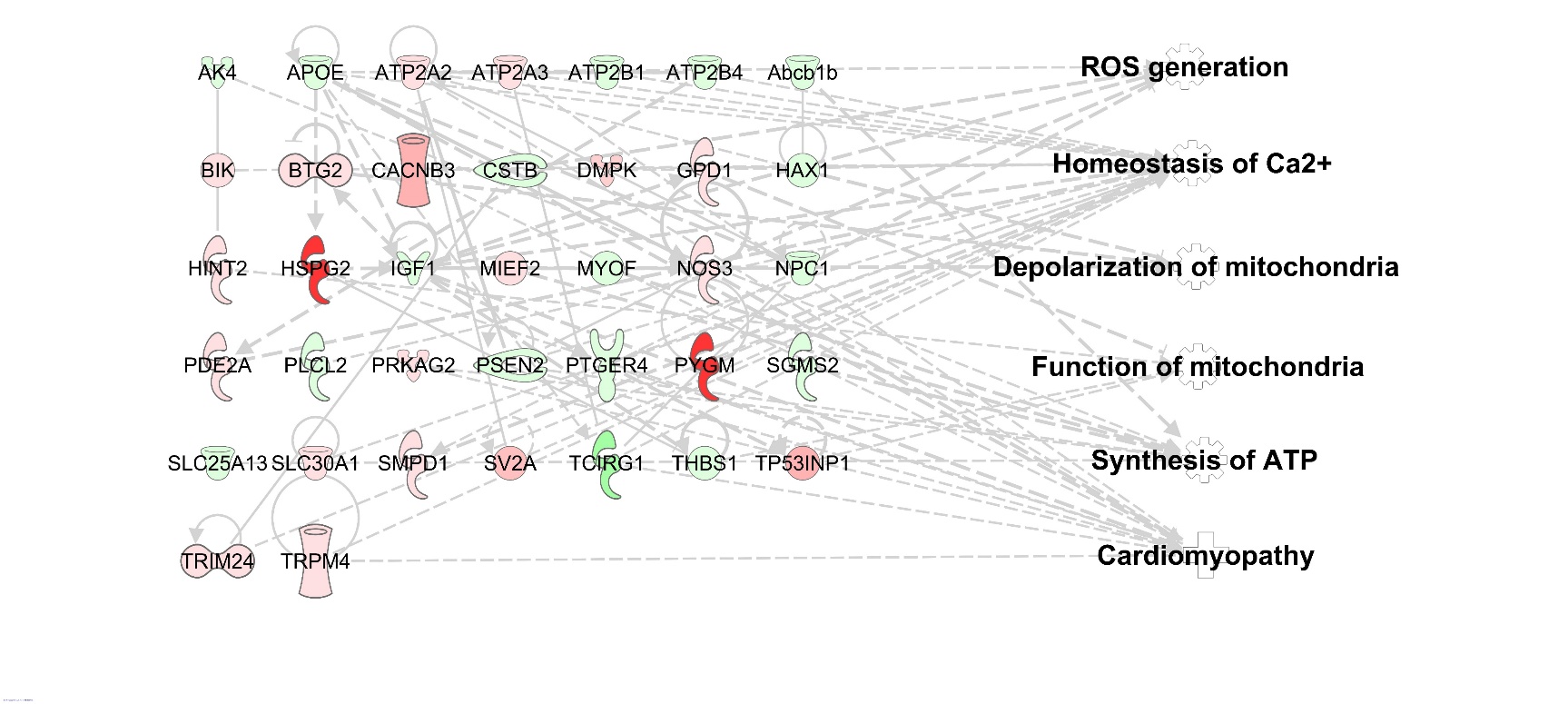
**

**Supplementary Figure 2 |** Functional analysis of the transcriptomic network of 100 µg/mL PM_2.5_-treated HL-1 cells using IPA. A fold change of ± 4 was used as the cut-off value. Red and green areas indicate genes whose expressions were upregulated and downregulated, respectively. Details for shape are provided in Supplementary Figure 1.


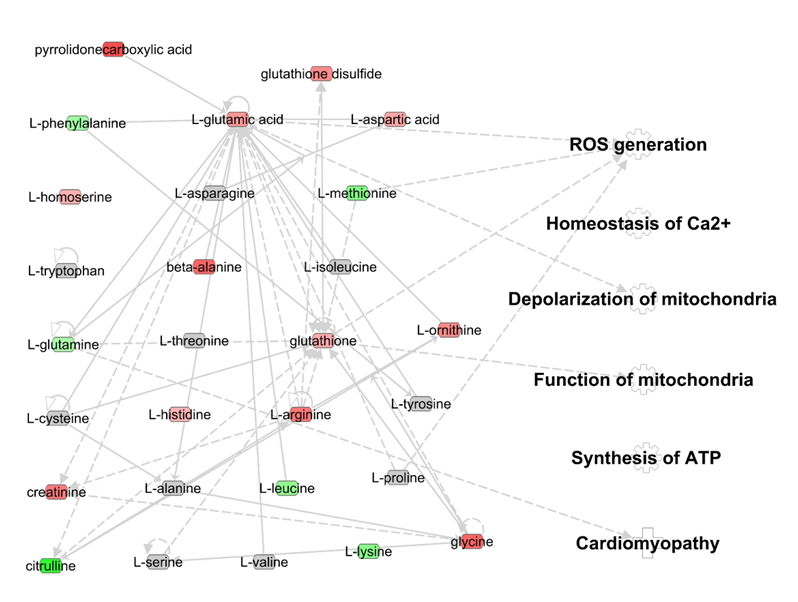


**Supplementary Figure 3 |** Functional analysis of the metabolic network of 10 µg/mL PM_2.5_-treated HL-1 cells. A fold change of ± 1.2 was used as the cut-off value. Red and green areas indicate metabolites whose levels were up- and downregulated, respectively. Details for shape and color are provided in Supplementary Figure 1 and Figure 2B.

**
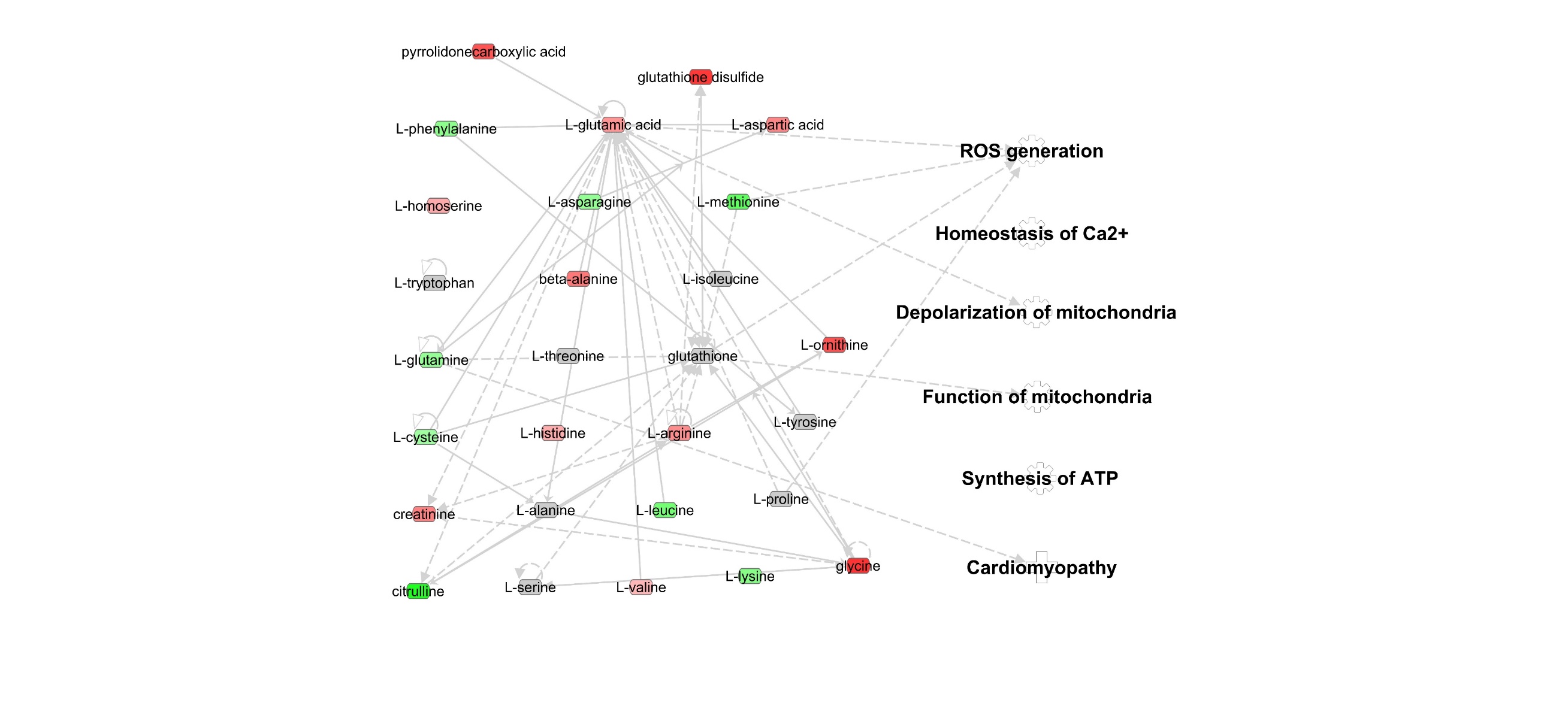
**

**Supplementary Figure 4 |** Functional analysis of the metabolic network of 100 µg/mL PM_2.5_-treated HL-1 cells. A fold change of ± 1.2 was used as the cut-off value. Red and green areas indicate metabolites whose levels were up- and downregulated, respectively. Details for shape and color are provided in Supplementary Figure 1 and Figure 2B.


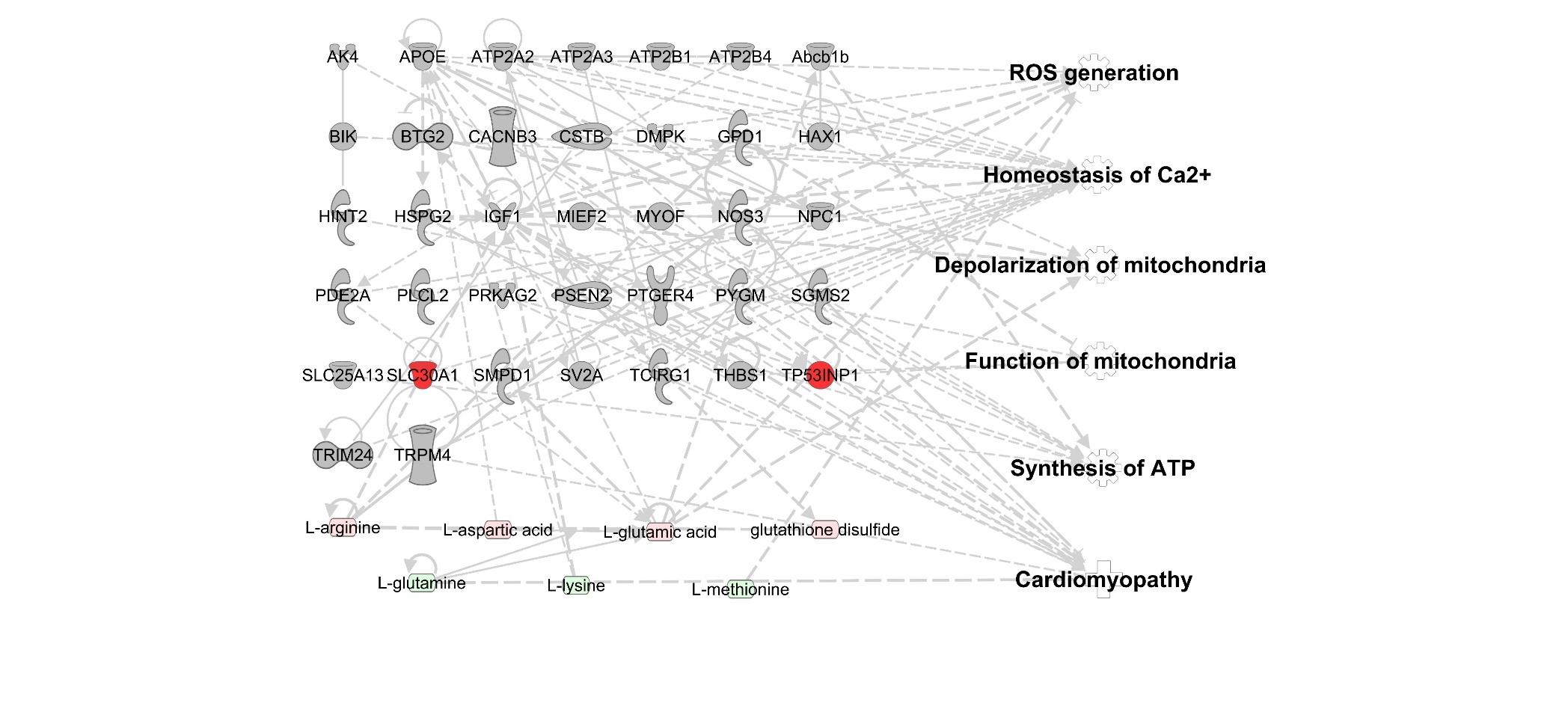


**Supplementary Figure 5 |** Functional analysis of the metabotranscriptomic network of 10 µg/mL PM2.5-treated HL-1 cells. Fold changes of ± 4 and ± 1.2 were used as cut-off values for genes and metabolites, respectively. Red and green areas indicate upregulated and downregulated proteins, respectively. Details for shape and color are provided in Supplementary Figure 1 and Figure 2B.

**
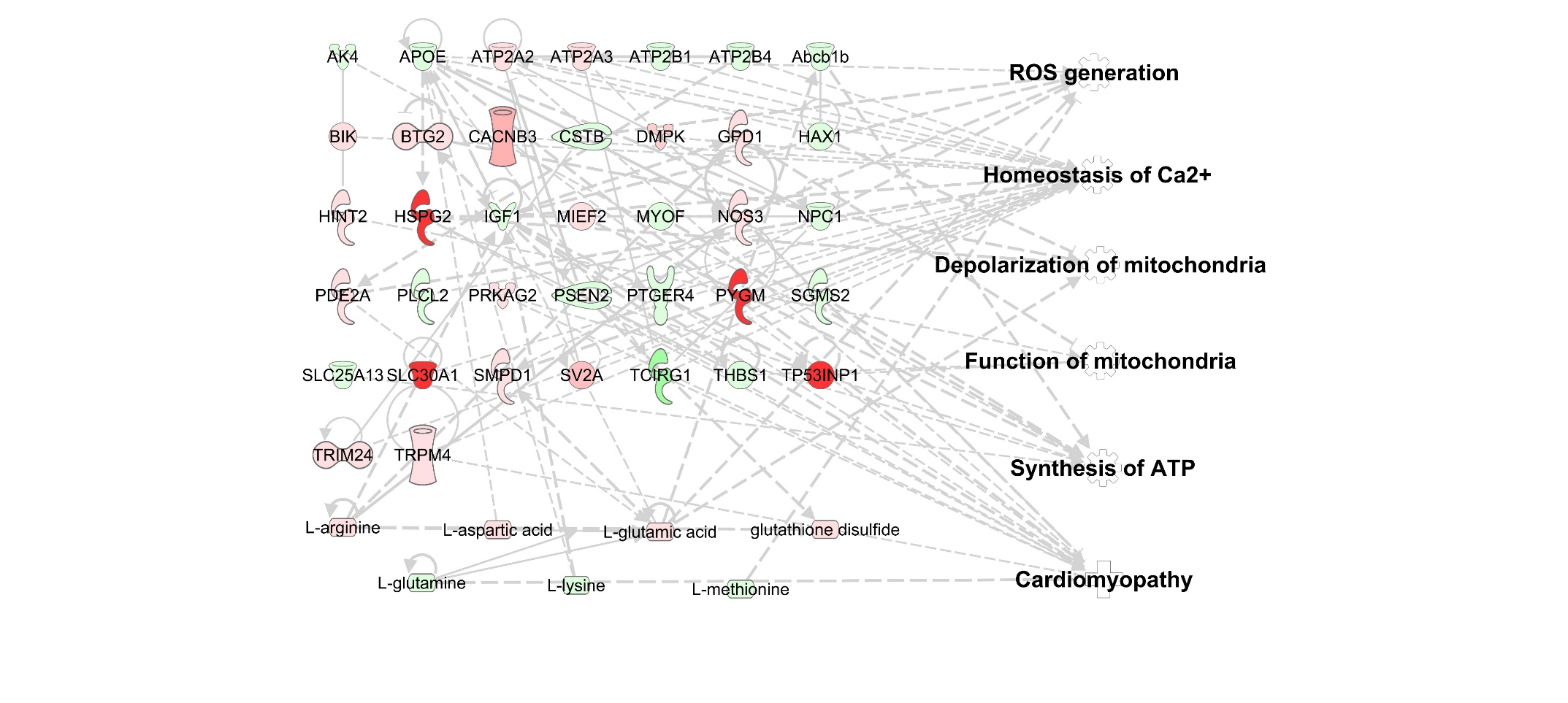
**

**Supplementary Figure 6 |** Functional analysis of the metabotranscriptomic network of 100 µg/mL PM2.5-treated HL-1 cells. Fold changes ± 4 for genes and ± 1.2 for metabolites were used as cut-off values. Red and green areas indicate up- and downregulated proteins, respectively. Details for shape and color are provided in Supplementary Figure 1 and Figure 2B.


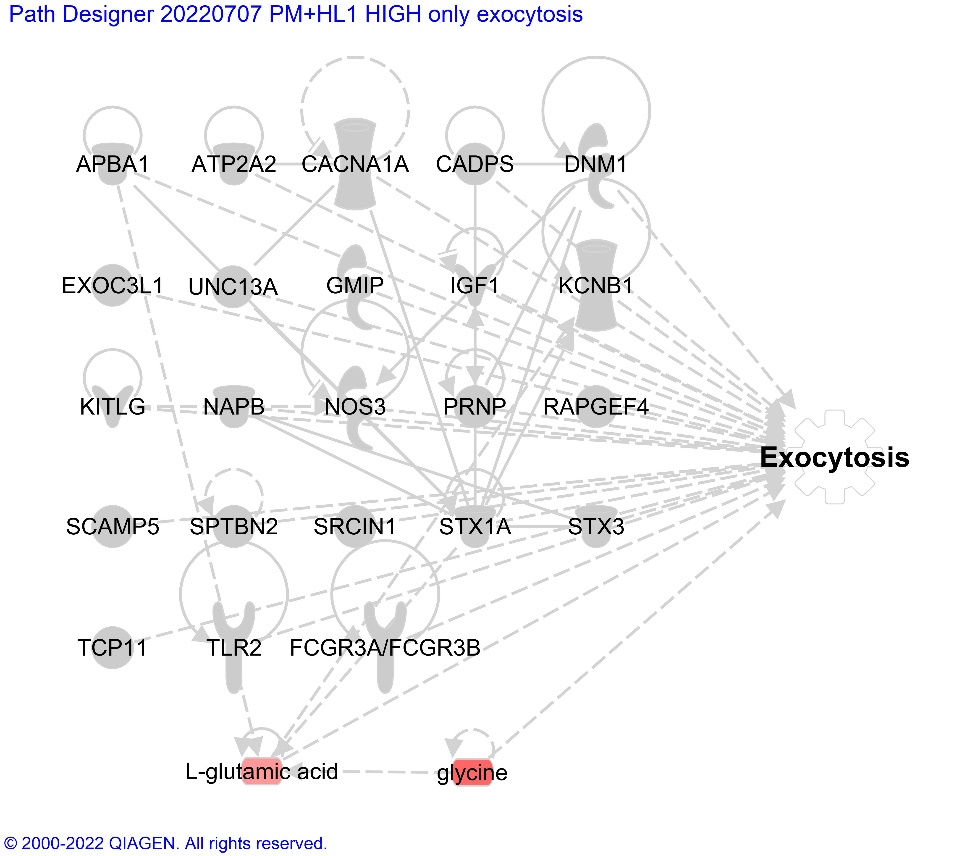


**Supplementary Figure 7 |** Functional analysis of the metabotranscriptomic network of 10 µg/mL PM2.5-treated HL-1 cells. Fold changes of ± 4 and ± 1.2 were used as cut-off values for genes and metabolites, respectively. Details for shape and color are provided in Supplementary Figure 1 and Figure 2B.


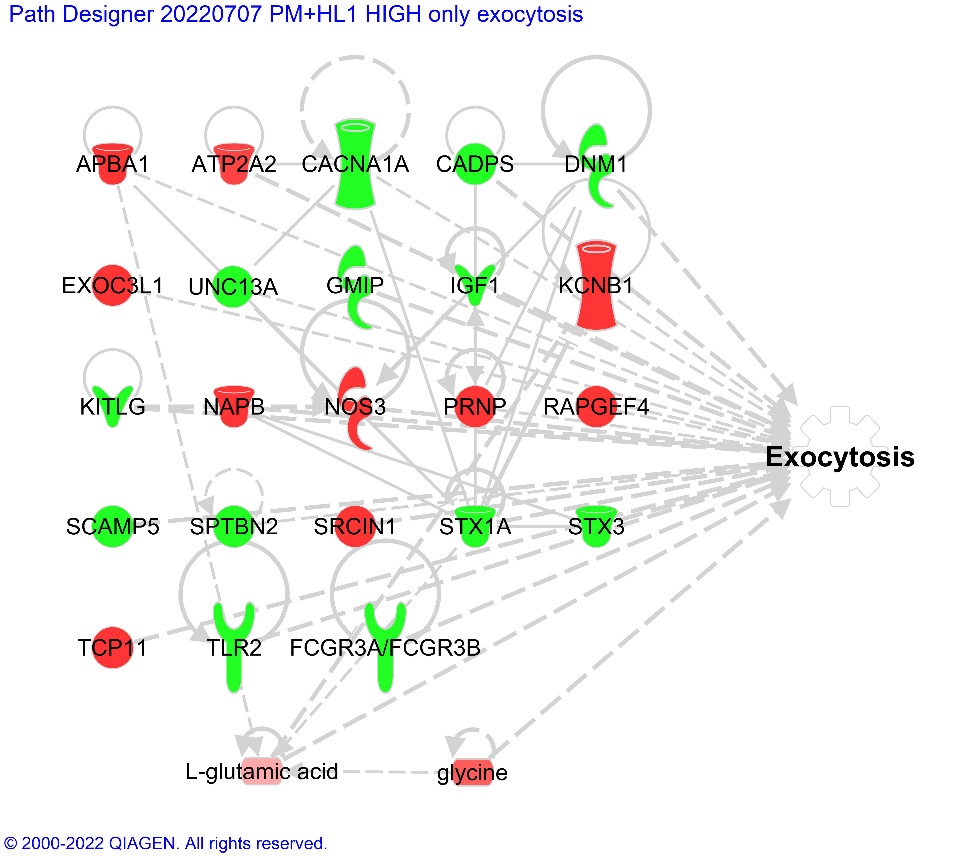


**Supplementary Figure 8 |** Functional analysis of the metabotranscriptomic network of 100 µg/mL PM2.5-treated HL-1 cells. Fold changes of ± 4 and ± 1.2 were used as cut-off values for genes and metabolites, respectively. Details for shape and color are provided in Supplementary Figure 1 and Figure 2B.


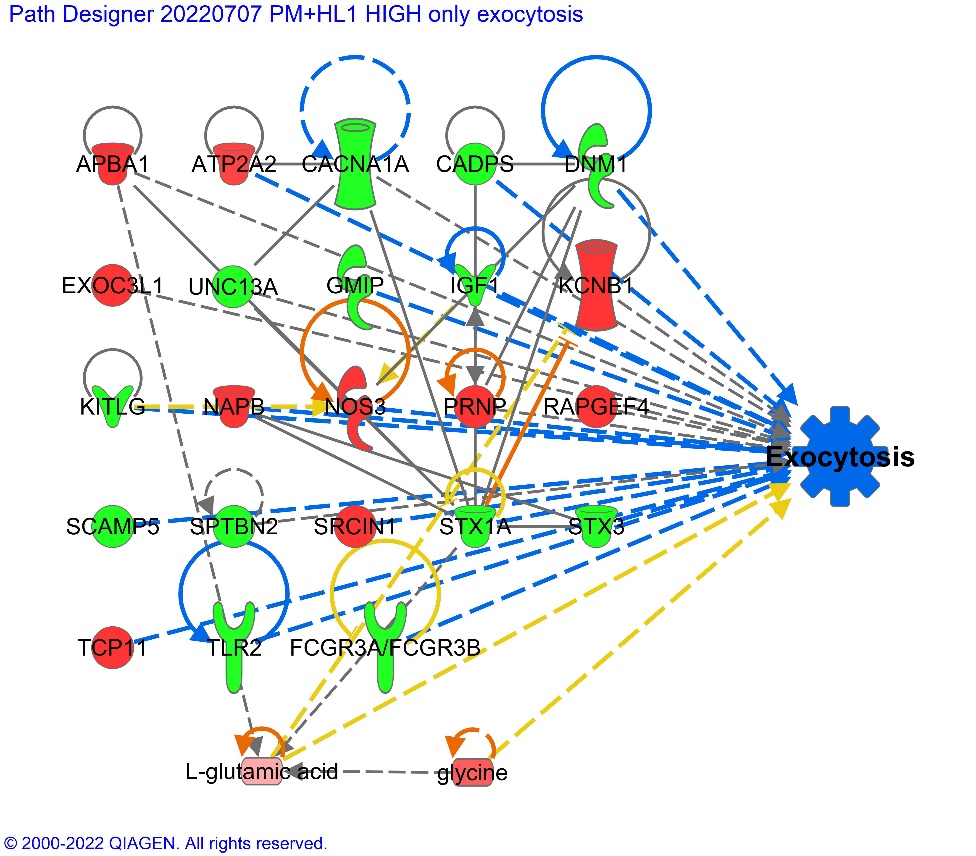


**Supplementary Figure 9 |** Functional analysis of the metabotranscriptomic network with the prediction of 100 µg/mL PM2.5-treated HL-1 cells. Fold changes of ± 4 and ± 1.2 were used as cut-off values for genes and metabolites, respectively. Details for shape and color are provided in Supplementary Figure 1 and Figure 2B.


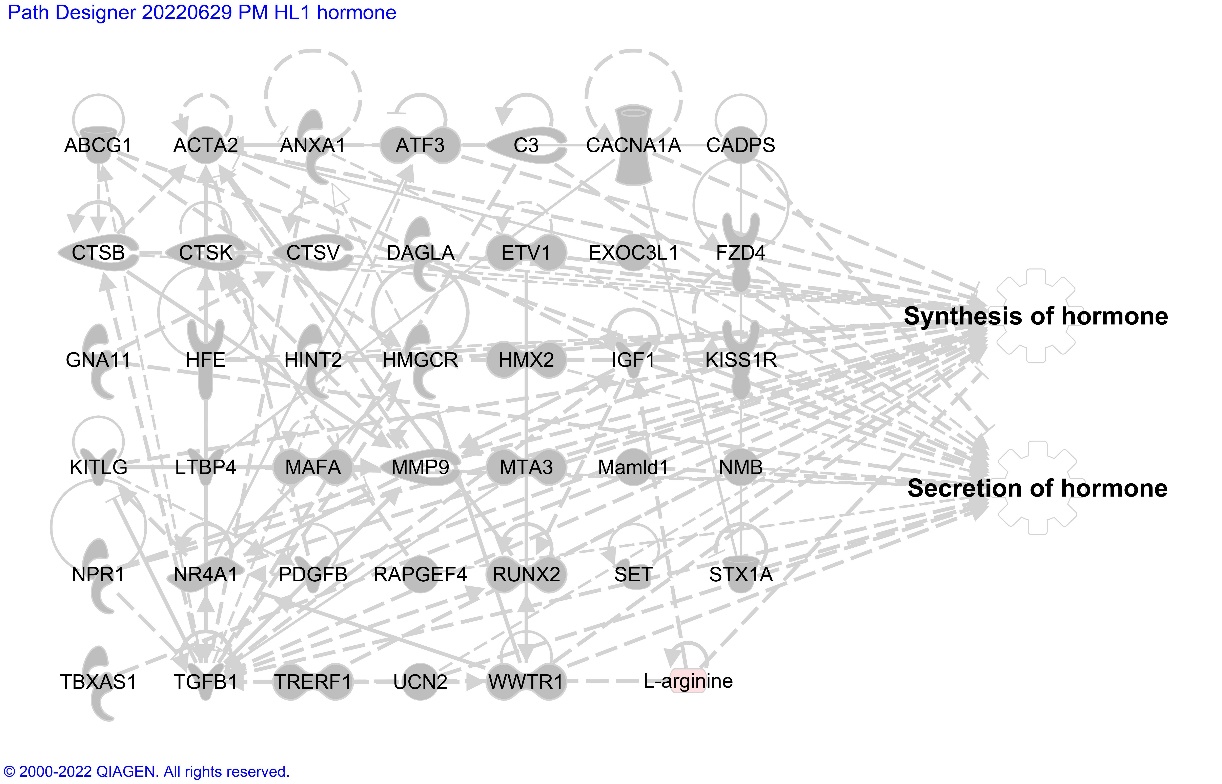


**Supplementary Figure 10 |** Functional analysis of the metabotranscriptomic network of 10 µg/mL PM2.5-treated HL-1 cells. Fold changes of ± 4 and ± 1.2 were used as cut-off values for genes and metabolites, respectively. Details for shape and color are provided in Supplementary Figure 1 and Figure 2B.


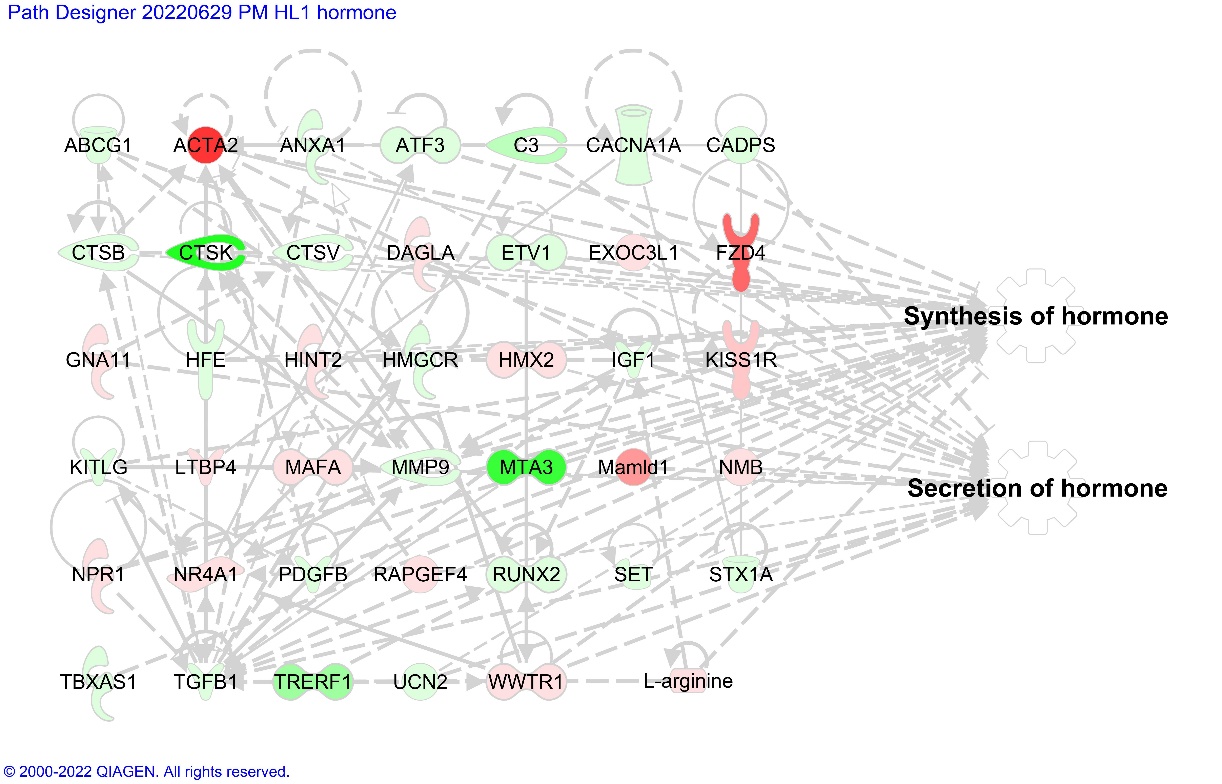


**Supplementary Figure 11 |** Functional analysis of the metabotranscriptomic network of 100 µg/mL PM2.5-treated HL-1 cells. Fold changes of ± 4 and ± 1.2 were used as cut-off values for genes and metabolites, respectively. Details for shape and color are provided in Supplementary Figure 1 and Figure 2B.


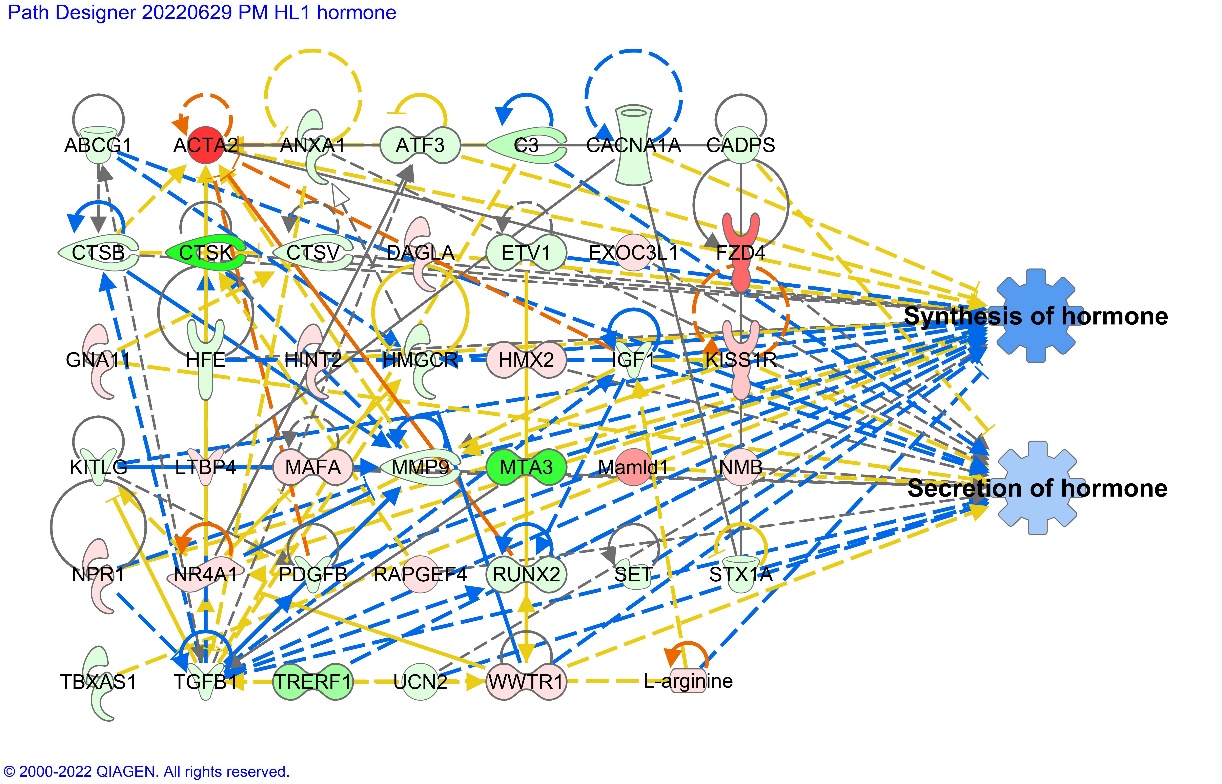


**Supplementary Figure 12 |** Functional analysis of the metabotranscriptomic network with the prediction of 100 µg/mL PM2.5-treated HL-1 cells. Fold changes of ± 4 and ± 1.2 were used as cut-off values for genes and metabolites, respectively. Details for shape and color are provided in Supplementary Figure 1 and Figure 2B.
